# Morphometric classification of kangaroo bones reveals paleoecological change in northwest Australia during the terminal Pleistocene

**DOI:** 10.1038/s41598-022-21021-w

**Published:** 2022-10-29

**Authors:** Erin Mein, Tiina Manne, Peter Veth, Vera Weisbecker

**Affiliations:** 1 School of Social Science, The University of Queensland, St Lucia, Australia; 2 Australian Research Council Centre of Excellence for Australian Biodiversity and Heritage, University of Wollongong, Wollongong, Australia; 3 Max Plank Institute for Geoanthropology, Jena, Germany; 4 School of Social Sciences, University of Western Australia, Crawley, Australia; 5 College of Science and Engineering, Flinders University, Bedford Park, Australia

**Keywords:** Biogeography, Palaeoecology, Archaeology, Palaeontology, Anthropology

## Abstract

Specimen identification is the backbone of archeozoological research. The challenge of differentiating postcranial skeletal elements of closely related wild animals in biodiverse regions can prove a barrier to understanding past human foraging behaviours. Morphometrics are increasingly being employed to classify paleozoological animal remains, however, the potential of these methods to discriminate between wild animal groups has yet to be fully realised. Here we demonstrate the applicability of a traditional morphometric approach to taxonomically classify foot and ankle bones of kangaroos, a large and highly diverse marsupial family. Using multiple discriminant analysis, we classify archaeological specimens from Boodie Cave, in northwest Australia and identify the presence of two locally extinct macropod species during the terminal Pleistocene. The appearance of the banded hare-wallaby and northern nail-tail wallaby in the Pilbara region at this time provides independent evidence of the ecological and human responses to a changing climate at the end of the last Ice Age. Traditional morphometrics provides an accessible, inexpensive, and non-destructive tool for paleozoological specimen classification and has substantial potential for applications to other diverse wild faunas.

## Introduction

The taxonomic identification of wild animal remains in archaeological deposits is crucial to our understanding of paleoecology and past food economies. Postcranial bones (including the skeleton but not the skull) make up a large proportion of paleozoological assemblages and are particularly informative for interpreting human interactions with animals because they provide information on carcass processing and body part utilisation. Delicate craniodental elements, with their wealth of taxonomic information, are often fragmented and dental morphology can become obscured through wear. Researchers often rely on postcrania to understand taxonomic diversity and human subsistence behaviour. However, in regions with high biodiversity such as Australasia, Southeast Asia, South America and the Neotropics, differentiating animals via postcranial bone is challenging owing to the large numbers of closely related and morphologically similar species.

The quantification of bone shape presents itself as a useful solution to taxon differentiation in biodiverse deposits. Morphometric approaches are routinely used in biology to describe evolutionary, phenotypic and functionally adaptive traits^1–4^. Archaeologists have long recognised the utility of a quantitative approach to identifying and exploring shape variation in archeozoological bone, particularly skulls and teeth^5,6^. However, such approaches have largely focused on differentiating morphologically similar domesticates such as sheep and goats^7–9^, understanding the morphological implications of the domestication process^10–12^ and describing economic and culturally driven changes in domesticate body size and skeletal shape^13–15^. In contrast, differentiation between wild species in biodiverse deposits still mostly relies on qualitative diagnostic criteria and physical reference collections. Quantitative approaches would advance Quaternary palaeozoological research, particularly where poor organic preservation does not permit the application of proteomic or molecular methods of identification^16–18^.

Accessibility is another major hurdle for the adoption of morphometrics in paleozoological research. Geometric morphometrics is a more powerful method for differentiating closely related species but is more complicated and specialised than traditional morphometrics which is more widely accessible^7,12^. Functional morphometrics demonstrate that postcranial bone shape of closely related wild taxa can substantially differ in biomechanical adaptation^19–21^. In Australia, functional analyses of marsupial postcrania using linear measurements have been very successful^22–24^, suggesting that a traditional morphometric approach could also produce an accessible and efficient means of taxonomically classifying marsupial postcrania in archaeological deposits.

Kangaroos of the superfamily Macropodoidea (macropods) are one of the most diverse marsupial groups in Australasia and are a particularly important group for paleozoologists because of their ecological and behavioural diversity. However, researchers are often hesitant to identify macropod postcranial bone beyond the family level owing to the morphological similarity between taxa^25^. Over 40 extant macropod species inhabit Australasia, ranging from under 1 kg to over 90 kg, at least eight species are recently extinct and a number of species are only known paleontologically^26^. These herbivores have played an important role in regional ecologies and Indigenous food economies and belief systems. They occupy every bioregion across Australasia and vary in their social behaviour, feeding and predator avoidance strategies. Understanding macropod diversity would provide information on local habitat structure, paleoenvironmental change and human hunting and resource management strategies in the past.

Current paleozoological interpretations of macropod remains are symptomatic of the wider challenges of dealing with a biodiverse radiation of species. Macropod postcranial remains which cannot be taxonomically identified are often differentiated into small, medium and large size categories^27–29^. Size is clearly an important discriminating characteristic between taxa, but broad size categories without additional taxonomic information have limited use in answering either paleontological or anthropological research questions. The quantification of specimen size may provide more precision for distinguishing between diverse wild species, including macropods, but requires clear and replicable protocols and the collation of comprehensive metric reference data.

Here we apply an easy-to-replicate method and R code, designed for low-cost linear measurements, to quantitatively compare macropod pes (foot and ankle) bones. We quantify pes bone size and shape to explore variation between macropod species and apply discriminant analysis to taxonomically classify unknown specimens. Tarsal bones and fourth metatarsals were chosen because individuals have only two of each bone (a left and a right) making them good candidates for providing unbiased quantification of minimum number of individuals (MNI) and examining taxonomic abundance^8^. Mammalian feet and ankles contain much locomotor information and a range of evolution-focused studies have successfully identified functionally adaptive variation between some macropod taxa, making them ideal candidates for morphometric classification^22,23,30–32^. Pes bones are also composed of compact, dense bone and are consistently found in good condition in paleozoological deposits.

We demonstrate the usefulness of our approach by classifying unknown specimens from an important archaeological site in Western Australia. Boodie Cave on Barrow Island contains the oldest known evidence for human occupation along an arid Pleistocene coastline in Australia^33^. Located 60 km off the Pilbara coast, the site lies on the edge of the northwest continental shelf and was occupied by people from ca. 51,000 to 7000 calibrated years before the present (cal BP). Formerly surrounded by sandy plains of the continental shelf, the island was isolated by rising sea levels in the mid-Holocene. Situated 20 degrees south, today the island has an arid subtropical climate and is located at the transition between the temperate weather systems to the south and tropical systems to the north, receiving unreliable annual rainfall from both the temperate winter rains and tropical summer monsoon^34^.

Archaeological evidence supports the deep and continuous cultural connections between the desert and the sea in the Pilbara region^33,35^. Regional patterns in faunal remains indicate a broad diet along this coastline during the terminal Pleistocene along with a shift towards marine resources as sea levels rose and coastline drew closer^28,33,36^. Terrestrial animals, particularly macropods, continued to play an important role in these Indigenous maritime-desert economies. Regional cave deposits and historical accounts provide evidence that, until the late nineteenth century, the mammalian fauna of the Pilbara bioregion was much more diverse than is seen today^37,38^. Understanding taxonomic diversity within late Quaternary cave deposits therefore provides an important independent line of evidence for ecological change and allows us to develop a more nuanced understanding of Indigenous adaptations to climatic and environmental changes in northwest Australia over the last 50,000 years.

## Materials and methods

Modern specimens from the Australian Museum (AM), Australian National Wildlife Collection (ANWC), Museum and Art Gallery of Northern Territory (MAGNT), the Queensland Museum (QM), the South Australian Museum (SAM), the Western Australian Museum (WAM), and the University of Queensland (UQ) Zooarchaeology Laboratory were used as a training dataset. The training dataset was composed of a total of 506 skeletal elements from eight genera and 17 species (Supplementary Table S1). Macropods that currently inhabit the Pilbara and neighbouring bioregions were included to account for potential biogeographical changes over time. The bridled nail-tail wallaby (*Onychogalea fraenata*), which does not occur in northwest Australia, was included to increase our sample size and act as a proxy for the small, extinct crescent nail-tail wallaby (*Onychogalea lunata*). Specimens of rare or extinct species could not be included in this study as their postcranial remains are rarely curated in museum collections.

Males and females along with juveniles and subadults were included in the training dataset to best reflect real-world paleozoological assemblage composition. An estimation of age was undertaken for the modern specimens as data on age at death was not available for each. Age was estimated via molar eruption stage rather than epiphyseal fusion stage which remains poorly understood due to the indeterminate growth patterns of many macropodids^39^. Bettong specimens were classed as adult when the permanent third premolar and all four molars were present^40–42^. Other macropods were classed as adults if all four molars were erupted^43^, as subadults if the second molar was fully erupted with the third premolar and third molar at least partially erupted^44–48^. All others were classed as juvenile.

A total of 13 macropod astragali, seven calcanea and six fourth metatarsals from the Boodie Cave assemblage were compared to the training dataset. This includes specimens from stratigraphic units (SU) one to seven, dating from ca. 50,000 cal BP (SU7) to the present day (SU1) (Supplementary Table S2, Supplementary Fig. S10).

### Traditional morphometric protocol

A total of 12 measurements were taken on the astragalus (Supplementary Fig. S11 and Table S3), 24 on the calcaneus (Supplementary Fig. S12 and Table S4) and 7 on the fourth metatarsal (Supplementary Fig. S13 and Table S5). Nomenclature for anatomical features follows Szalay^32^, including the term ‘astragalus’ for the tarsal bone which is also often termed ‘talus’. Each measurement was taken three times, non-consecutively by the same operator (EM) using digital callipers, and the mean of the three measurements was used in all further analyses. The standard deviation between repeat measurements tended to be proportionally higher on smaller bones. This was expected given very small bones are difficult to measure. Despite this, intraclass correlations between all repeat measurements were very high (≥ 0.9) indicating intraoperator measurement error was unlikely to impact further analyses (see Supplementary Table S6)^49^.

A step-wise test for measurement redundancy was performed on the astragali and calcanea to reduce the dimensionality of the data and meet the within-group sample size criteria for discriminant analysis. This aimed to identify measurements which could be removed without affecting the ordination of skeletal element shape. A high degree of redundancy was found between all measurements, therefore measurements which were rarely preserved on our archaeological specimens such as those incorporating the epiphysis, were excluded from further analyses. A total of seven measurements on the astragalus and metatarsal and nine on the calcaneus were used in all subsequent analyses (see Supplementary Tables S3, S4 and S5 online).

### Quantify and describe bone size and shape variation

The geometric mean was used as a proxy for bone size rather than individual measurements or ratios which can be influenced by allometry^50^. Categorisation of macropod species into size groups was done visually by plotting element size by species. All subsequent analyses were performed within the relevant size groups to compare only those taxa which overlap in size and would be difficult to visually discriminate. As age and sexual dimorphism can increase intraspecific size variation and cause overlap between our size groups any taxa which overlapped two size groups were included in both.

We examined how bone shape varies proportionally between similar sized taxa using Principal Components Analysis (PCA) on size-free shape variables. Shape variables were transformed using the log-shape ratio method (log10(measurement / geometric mean)) to remove size but retain allometry^51^. If taxa were clearly differentiated in the main variation of the PCA, we examined the loadings of each principal component (PC) to understand the regions of greatest shape variation at an inter and intraspecific level. Multivariate analysis of variance was performed following Claude^50^ to test the variance of element shape with size (indicating allometry or disproportionate change with size), age and sex where this information was available.

The size of archaeological specimens was assessed against the training data and each specimen allocated to a size group. The size-free shape of archaeological specimens was compared to the training dataset in the main variation of the PCAs.

### Discriminant analysis

We applied multiple linear discriminant analysis (LDA) to our training dataset to test whether our measurements could accurately predict the genus of unknown specimens and thus provide a quantitative method for taxonomically classifying paleozoological bones. Classification to species was not undertaken due to small sample sizes at the species level. As specimen size provides discriminating information, particularly for medium and large-bodied macropods, we performed a cross-validated LDA on bone form (size + shape) within each size category. To reduce the potential for overfitting, the discriminant functions were evaluated using a leave-one-out (jacknife) method of cross validation with 1000 iterations. The probability of specimens being correctly reclassified to the correct taxonomic group (the hit ratio) was then calculated.

Given the relatively small and uneven within-group sample sizes and heteroscedasticity in the training dataset, the accuracy of each hit ratio was assessed using three criteria: maximum chance criterion (Cmax), proportional chance criterion (Cpro) and a Press Q test^52–54^. The thresholds for each hit ratio were set at 1.25 times the Cmax and Cpro following Hair et al.^55^.

### Taxonomic classification of paleozoological specimens

Archaeological specimens were classified to genus using the discriminant functions developed with the training dataset. Archaeological specimens were introduced into the discriminant analyses as unknowns and posterior probabilities were calculated for each specimen. Given the antiquity of the paleozoological deposits at Boodie Cave some potential exists for unknown, extinct taxa which are not included in our training sample. We would expect specimens of unknown genera to fall outside the distribution of taxa in our LDA plots. Specimen size and size-free shape should be assessed independently as supporting evidence for the presence of unknown taxa.

All analyses were carried out in R (v.4.1.0) using the packages MASS^56^, car^57^, Hmisc^58^, vegan^59^, rstatix^60^, MVN^61^ and psych^62^.

## Results

### Quantifying bone size

**Figure 1:**
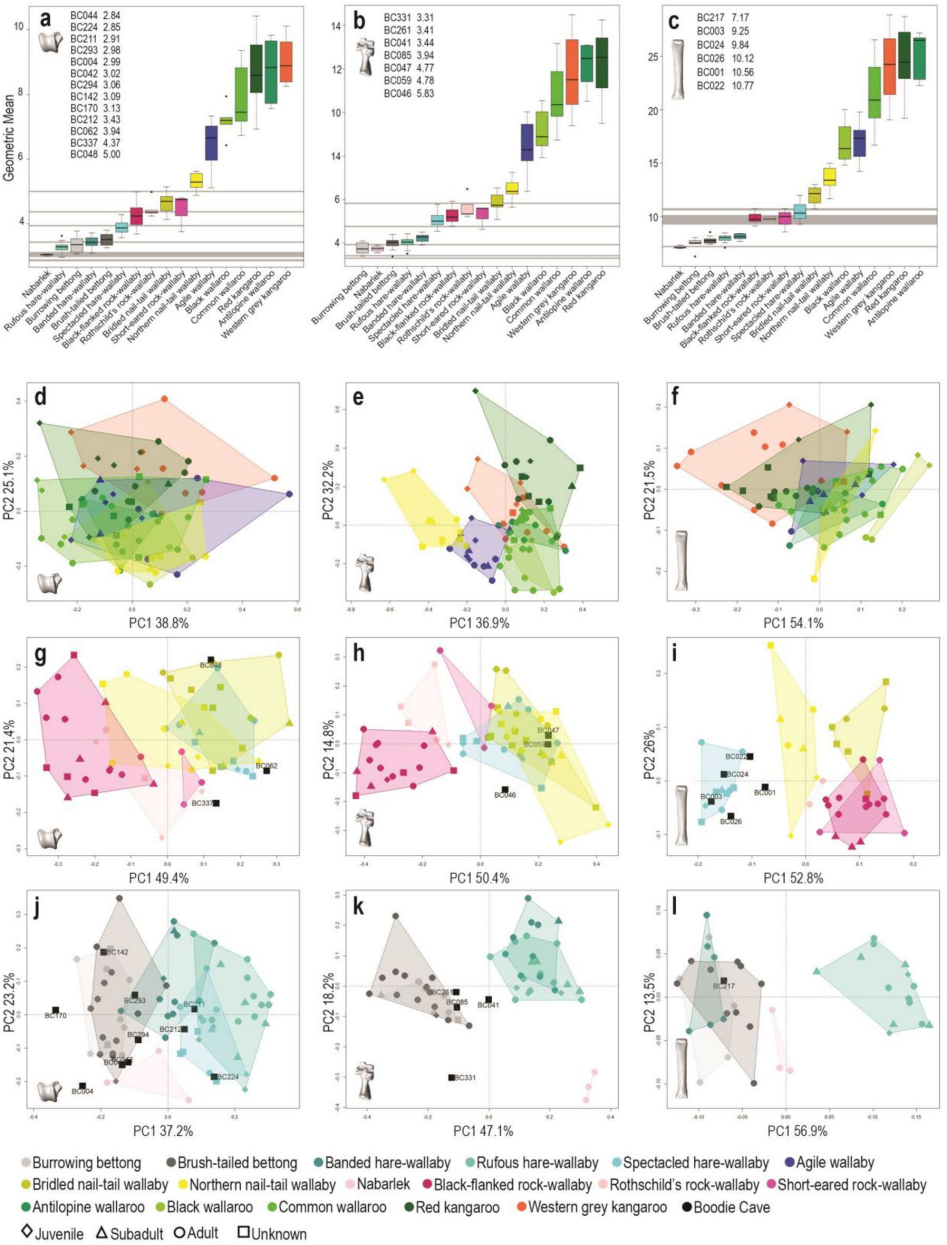
Size and shape of macropod pes bones by species. (**a–c**) Size of macropod astragali (left), calcanea (middle) and metatarsal IV (right) by species. Geometric mean of each archaeological specimen is listed from smallest to largest, grey bars indicate size range of archaeological specimens compared to training dataset; (**d–f**) PCA of large macropod astragali (left), calcanea (middle) and fourth metatarsal (right); (**g–i**) PCA of medium macropod astragali (left), calcanea (middle) and fourth metatarsal (right); (**j–l**) PCA of small macropod astragali (left), calcanea (middle) and fourth metatarsal (right). Figure generated with R (v.4.1.0) (www.cran.r-project.org) and Adobe Indesign (v16.4) (www.adobe.com).

Using the geometric mean of measurements for each bone allowed us to empirically divide the modern training dataset into the commonly used small, medium and large body-size categories. The large size group included red kangaroos (*Osphranter rufus*), western grey kangaroos (*Macropus fuliginosus*), common wallaroos (*Osphranter robustus*), antilopine wallaroos (*Osphranter antilopinus*), black wallaroos (*Osphranter bernardus*) and agile wallabies (*Notamacropus agilis*) (Fig. 1). The medium group included spectacled hare-wallabies (*Lagorchestes conspicillatus*), three rock-wallabies (*Petrogale lateralis*, *Petrogale brachyotis* and *Petrogale rothschildi*), northern nail-tail wallabies (*Onychogalea unguifera*) and bridled nail-tail wallabies (*Onychogalea fraenata*). The small group included burrowing (*Bettongia lesueur*) and brush-tailed (*Bettongia penicillata*) bettongs, banded hare-wallabies (*Lagostrophus fasciatus*), rufous hare-wallabies (*Lagorchestes hirsutus*) and the Nabarlek (in Bininj Kunwok dialects) (*Petrogale concinna*). Pes bones of northern nail-tail wallabies overlapped both medium and large size groups, and astragali of spectacled hare-wallabies overlapped the small and medium size groups.

Most specimens from Boodie Cave fell within the small and medium size groups of the modern species, although four specimens (BC044, BC211, BC224 and BC331) were smaller than any modern specimen (Fig. 1). In many cases, the taxonomy of the archaeological specimens could be narrowed down to three or four species simply by quantifying and comparing bone size. Size has greater discriminating power in the medium and large size categories where species have an almost continuous gradation in size from smallest to largest (Fig. 1). Comparison of size is less useful in the small size category as small macropod species exhibit the most constrained size range and highest degree of overlap.

### Shape variation between macropod species

The below describes PCA on size-free shape variables, focusing on the first two Principal Components (PC1 and 2) which together reflect the main variation of pes bone shape between macropods when size overlaps. PCA is agnostic to group membership^63^ and therefore not suitable to statistically differentiate groups of interest. However, the first few PCs are summaries of the greatest amount of variation that is independent of other PCs, making PC1/PC2 plots suitable for approximating the variation that visual inspection would also reveal. The below summarises the results; specific morphological differences between the pes bones of different macropod species in each size group are described in detail in the supplementary materials (see Supplementary Note online).

#### Large macropods

When isometric size is removed, the shape of large macropod astragali and fourth metatarsals overlaps substantially in the main variation of the PCA (Fig. 1). In contrast, the calcaneal shape appears to be much more distinct between large species. Consistent trends for shape variation between immature and adult large macropods can be observed in the main variation and, when tested, the shape of all three pes bones does vary significantly with age (p < 0.001) (Supplementary Table S7). Examination of the PC loadings reveals that immature large macropods tend to have astragali with longer talar necks, calcanea with a deeper calcaneocuboid step and more dorsoventrally slender fourth metatarsals. However, as these characteristics also contribute towards interspecific shape variation, this effectively increases the overlap in pes bone shape between species. Significant differences in fourth metatarsal shape (F = 7.255, p = 0.003), most likely allometric, were observed between common wallaroos from the mainland and the dwarf subspecies that inhabit Barrow Island (Supplementary Table S7). Large-bodied macropods exhibit a high degree of sexual dimorphism with dominant males substantially larger than females. Despite this, sex did not have a significant effect on intraspecific bone shape in any species where sample size permitted testing (Supplementary Table S7). Shape overlap between taxa and intraspecific variation suggests that large macropod astragali and fourth metatarsals would be particularly difficult to visually identify, and caution should be used when assuming morphological differences are taxonomically diagnostic.

#### Medium macropods

Rock-wallabies (genus *Petrogale*) vary substantially in shape from the spectacled hare-wallaby and both nail-tail wallabies (genus *Onychogalea*) across all three pes bones (Fig. 1). Some difference in ankle bone shape may occur between the three species of rock-wallaby in our training dataset, but a larger sample is needed to better understand shape variation amongst rock-wallaby species. The astragali and calcanea of spectacled hare-wallabies and nail-tail wallabies (genus *Onychogalea*) are very similar in shape but clear differences are observed in the shape of their fourth metatarsals along PC1. Surprisingly, the metatarsal shape of northern nail-tail wallabies and bridled nail-tail wallabies does not overlap and is clearly separated along PC1.

Bone shape variation between medium sized species occurs largely along PC1, while PC2 contains much of the intraspecific shape variation in this size class (Fig. 1). Intraspecific shape variation is therefore likely to have a less confounding effect on the visual differentiation of medium macropods than large macropods. Immature medium macropods tend to score negatively along PC2 for each pes bone, although only calcaneal shape varies significantly with age amongst medium macropods (F = 3.609, p = 0.006). However, a weak signal for allometry is observed in the calcanea of spectacled hare-wallabies (F = 8.321, p = 0.01) (Supplementary Table S8). Spectacled hare-wallabies are not sexually dimorphic in body size but may be subject to similar regional size variations as the closely related rufous hare-wallaby^64^. A larger sample size is needed to test the causes of allometry in this species. It should be noted that visual shape differences in the pes bones of spectacled hare-wallabies may be related to intraspecific size variation rather than diagnostic and should be treated with caution.

Medium sized specimens from Boodie Cave appear similar in shape to spectacled hare-wallabies and nail-tail wallabies (genus *Onychogalea*) in the main variation of the PCAs (Fig. 1).

#### Small macropods

Spectacled and rufous hare-wallabies (genus *Lagorchestes*) appear to have distinctly shaped pes bones from the bettongs (genus *Bettongia*) and are clearly separated along PC1 (Fig. 1). All three pes bones of the burrowing and brush-tailed bettongs are likely visually indistinguishable as they overlap almost entirely in the main variation of the PCAs. Astragali of spectacled and rufous hare-wallabies are also very similar, overlapping substantially in the main variation. Banded hare-wallaby (genus *Lagostrophus*) calcanea are similar in shape to rufous hare-wallabies but their fourth metatarsals are more similar to bettongs. Astragalus shape of banded hare-wallabies appears to contain a mixture of bettong and *Lagorchestes* genus shape characteristics.

Intraspecific shape variation is again largely observed along PC2 and juvenile specimens in each species tend to score negatively along this eigenvector. Despite this trend, only the fourth metatarsal shape varied significantly with age for small-bodied macropods (F = 3.896, p = 0.007) (Supplementary Table S9). Small-bodied macropods do not exhibit substantial sexual dimorphism and sex did not significantly affect intraspecific pes bone shape. Significant differences in the shape of astragali (F = 21.982, p =  < 0.001) and calcanea (F = 5.58, p = 0.009) between mainland and island dwelling rufous hare-wallabies are observed although, interestingly these do not appear to be allometric (Supplementary Table S9). Intraspecific variation in rufous hare-wallaby ankle bone morphology may be misleading and confound attempts to visually differentiate this taxon from other genera.

Two small astragali from Boodie Cave are similar in shape to both hare-wallaby genera (*Lagorchestes* and *Lagostrophus*) and a third plots within the rufous hare-wallaby morphospace. The remaining small specimens cluster closely to the bettong morphospace. Two astragali and two calcanea from Boodie Cave cluster close to the bettong morphospace but do not overlap with the modern specimens (Fig. 1).

### Discriminant analysis

**Figure 2:**
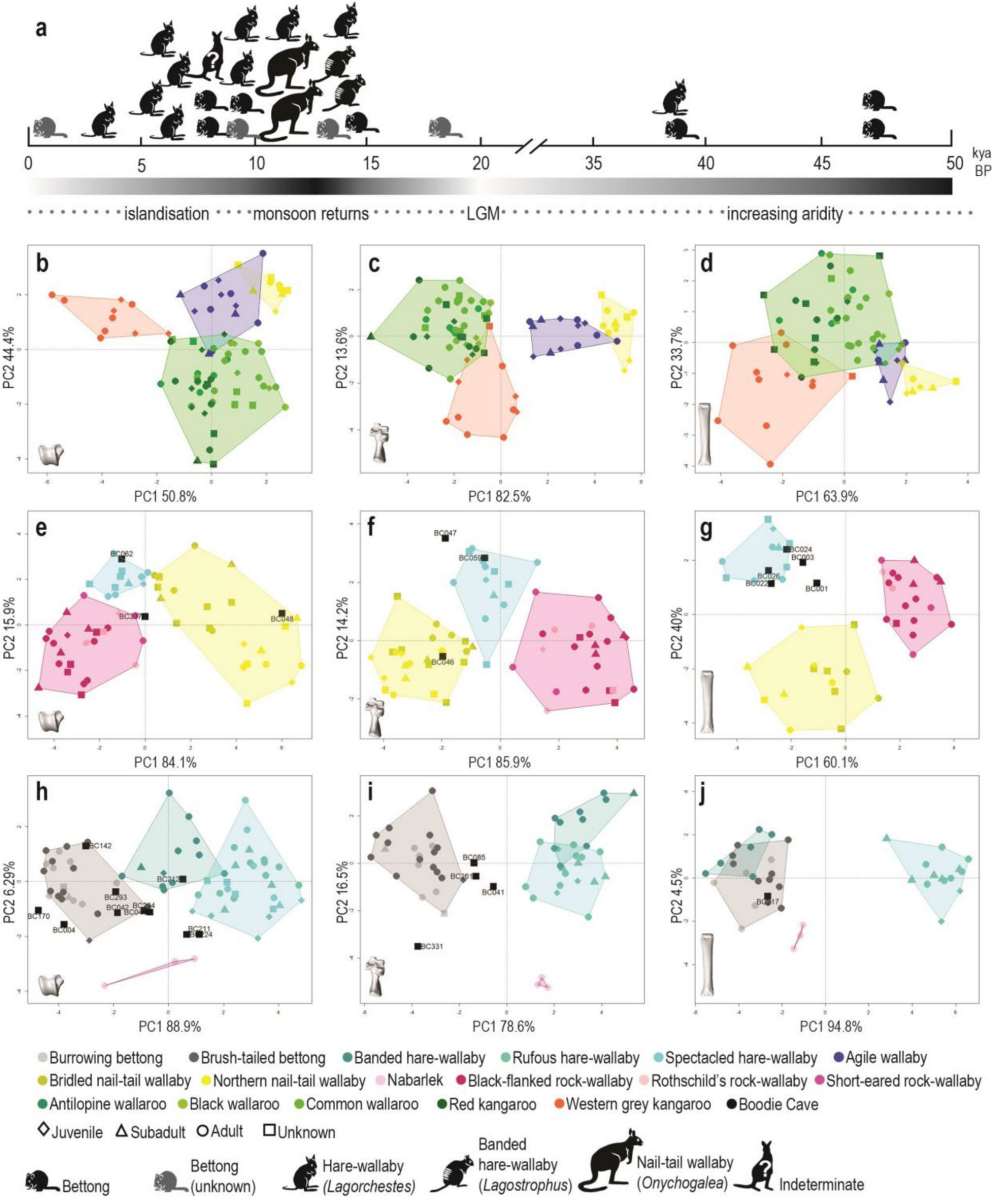
Taxonomic classification of macropod pes bones by discriminant analysis. (**a**) macropod genera in Boodie Cave over time; (**b–d**) classification of large macropod astragali (left), calcanea (middle) and fourth metatarsals (right) by genus; (**e–g**) classification of medium macropod astragali (left), calcanea (middle) and fourth metatarsals (right) by genus; (**h–j**) classification of small macropod astragali (left), calcanea (middle) and fourth metatarsals (right) by genus. Figure generated with R (v.4.1.0) (www.cran.r-project.org) and Adobe Indesign (v16.4) (www.adobe.com).

Plots of the main variation in each LDA illustrate a clear separation between genera in each size class (Fig. 2). The inclusion of size in the discriminant analysis allows for large macropod pes bones to be clearly differentiated to genera, despite the substantial overlap in shape observed in the PCAs. The discriminant scores of northern and bridled nail-tail wallabies are also well differentiated for each pes bone due to the differences in size between these two species (Fig. 2). Examination of the LD loadings indicate variation in pes bone form between genera is driven by complex combinations of linear measurements suggesting that a multivariate approach to morphometric classification is likely to be more robust than individual measurements or ratios (Supplementary Fig. S14).

The probability of unknown specimens being correctly reclassified by the discriminant functions (the hit ratio) was ≥ 0.9 for all bones in all size classes, except large and small fourth metatarsals (Table 1). Except for large macropod metatarsals, all hit ratios exceeded the thresholds of our test criteria (Table 1).

**Table 1:** Cross validated hit ratios for each skeletal element.

Pes bone	Hit ratio	*Cmax* + *25%*	*Cpro* + *25%*	*Press Q*
**Large macropods**				
Astragalus	**0.90**	0.73	0.50	177.32*
Calcaneus	**0.92**	0.69	0.46	188.46*
Metatarsal IV	0.74	0.74	0.51	85.41*
**Medium macropods**				
Astragalus	**0.93**	0.54	0.46	93.24*
Calcaneus	**0.90**	0.49	0.44	88.66*
Metatarsal IV	**0.95**	0.55	0.44	74.42*
**Small macropods**				
Astragalus	**0.94**	0.53	0.42	173.25*
Calcaneus	**0.90**	0.53	0.41	118.56*
Metatarsal IV	**0.79**	0.49	0.40	58.98*

Bold indicates a correct reclassification probability that exceeds the thresholds for all three test criteria.

*Significant at p = 0.1.

### Taxonomic classification of paleozoological specimens

Specimens from Boodie Cave were classified to genus by the discriminant functions developed with the training dataset. All but one specimen (BC337) could be confidently classified to a single genus. We did not attempt to classify specimens to species due to our small sample size, however an examination of the discriminant scores and specimen size provides good indication of species level identifications for some specimens.

A total of ten pes bones were classified as hare-wallabies in the genus *Lagorchestes* (Table 2). While *Lagorchestes* specimens dominate the early to mid Holocene period, specimens from this genus are found throughout the stratigraphic sequence at Boodie Cave (Fig. 2a). Comparison of specimen size with the training dataset indicates that both the larger spectacled hare-wallaby and smaller rufous hare-wallaby are present in the cave. Both species persist on Barrow Island today, inhabiting sandy dunes and spinifex (*Triodia*) grasslands^65^.

**Table 2:** Classification matrix for specimens from Boodie Cave.

Specimen ID	*Bettongia*	*Lagorchestes*	*Lagostrophus*	*Onychogalea*	*Petrogale*
**Medium astragali**					
BC048	NA	0.000	NA	**1.000**	0.000
BC062	NA	**0.998**	NA	0.001	0.001
BC337	NA	**0.486**	NA	0.132	0.382
**Small astragali**					
BC004	**1.000**	0.000	0.000	NA	0.000
BC042	**0.605**	0.000	0.247	NA	0.148
BC044	0.167	0.022	**0.647**	NA	0.164
BC142	**0.834**	0.000	0.166	NA	0.000
BC170	**1.000**	0.000	0.000	NA	0.000
BC211	0.000	**0.625**	0.112	NA	0.264
BC212	0.001	0.278	**0.721**	NA	0.001
BC224	0.001	**0.989**	0.003	NA	0.007
BC293	**0.991**	0.000	0.008	NA	0.000
BC294	**0.663**	0.022	0.258	NA	0.057
**Medium calcanea**					
BC046	NA	0.009	NA	**0.991**	0.000
BC047	NA	**0.997**	NA	0.003	0.000
BC059	NA	**0.999**	NA	0.001	0.000
**Small calcanea**					
BC041	**0.668**	0.330	0.001	NA	0.000
BC085	**0.998**	0.002	0.000	NA	0.000
BC261	**0.998**	0.002	0.000	NA	0.000
BC331	**1.000**	0.000	0.000	NA	0.000
**Medium fourth metatarsals**					
BC001	NA	**0.928**	NA	0.022	0.051
BC003	NA	**0.999**	NA	0.000	0.001
BC022	NA	**0.999**	NA	0.001	0.000
BC024	NA	**1.000**	NA	0.000	0.000
BC026	NA	**1.000**	NA	0.000	0.000
**Small fourth metatarsals**					
BC217	**0.965**	0.000	0.025	NA	0.010

Highest posterior probability for each specimen indicated in bold.

A total of 11 pes bones were classified as bettongs and are also found throughout the stratigraphic sequence and commonly co-occur with *Lagorchestes* specimens (Table 2, Fig. 2a). The bettong foot and ankle bones at Boodie Cave likely belong to the burrowing bettong, an arid zone specialist which is known to inhabit Barrow Island today. However, two astragali (BC004 and BC170) and two calcanea (BC041 and BC331) fall outside of the bettong morphospace in both the PCA and LDA and are smaller than modern bettongs in our training dataset (Figs. 1 and 2). The differences in shape and size suggest these four specimens may not belong to either the burrowing or brush-tailed bettong but may represent an unknown bettong taxon.

Four pes bones were classified to two taxa which do not occur in the Pilbara region today. Two astragali (BC044 and BC212) were classified as banded hare-wallaby (genus *Lagostrophus*) and an astragalus (BC048) and calcaneus (BC046) were classified as nail-tail wallaby (genus *Onychogalea*) (Table 2). Comparison of specimen size indicates BC046 and BC048 likely belonged to the northern nail-tail wallaby as they are substantially larger than would be expected for the now extinct crescent nail-tail wallaby which was closer to the spectacled hare-wallaby in size (Fig. 1). The nail-tail (*Onychogalea*) and banded hare-wallaby (*Lagostrophus*) ankle bones were recovered from the upper levels of stratigraphic unit five (SU5) dating to the terminal Pleistocene and early Holocene (Fig. 2a).

## Discussion

In Australasia, recent human impacts have fundamentally altered the distribution of many native species and caused the extinction of at least 34 mammal species^26^. Paleozoological assemblages therefore provide an important window onto the environmental and ecological context of Indigenous economies in both the deep and recent past. In many regions morphological identification of mammalian remains will continue to be necessary where environmental conditions rapidly degrade the DNA or collagen needed for molecular and proteomic identification^69^. Here we have demonstrated that a traditional morphometric approach can classify three macropod pes bones to genus with a high degree of accuracy. Morphometrics are also non-destructive and inexpensive compared to proteomic or DNA analysis.

We found that variation in pes bone form between macropod genera in all size groups is driven by complex combinations of linear measurements. Therefore, a multivariate approach to classification is likely to be more robust at differentiating taxa than individual measurements or ratios. The ordination of shape after removing isometric size demonstrates that the pes bones of small and medium macropods vary more substantially between species than those of large macropods and it may be possible to visually differentiate genera in these size groups. Large macropods exhibit substantial intraspecific and ontogenetic shape variation in their pes bones which means that observable shape differences between individual specimens may not be taxonomically diagnostic and should be treated with caution. Where sample size permitted testing, sexual dimorphism did not have a significant influence on pes bone shape and is therefore unlikely to introduce error into the classification of remains at the genus level. Despite the degree of size overlap between species, a quantitative comparison of size was useful for narrowing down the potential taxonomy of individual archaeological specimens. Rather than using individual measurements as overall size predictors, we recommend that the geometric mean be used as a proxy for specimen size because this reduces the potential confounding effects of intraspecific variation in size (and potential allometric effects) on individual measurements.

The high degree of redundancy in our measurement protocol for astragali and calcanea suggests that taxonomic identification of more fragmentary macropod pes elements may be possible using alternative combinations of measurements. Future research should investigate the potential for classifying fragmentary remains where data is missing, as this would substantially enhance the applicability of the method to archaeological assemblages which are often highly fragmented. Increasing the within-group sample sizes and expanding the training dataset to include all Australasian macropod species would also improve the accuracy and generalizability of our model^52^. However, it is important to note that comprehensive postcranial datasets of some species are difficult to obtain due to their limited presence in museum collections.

**Figure 3:**
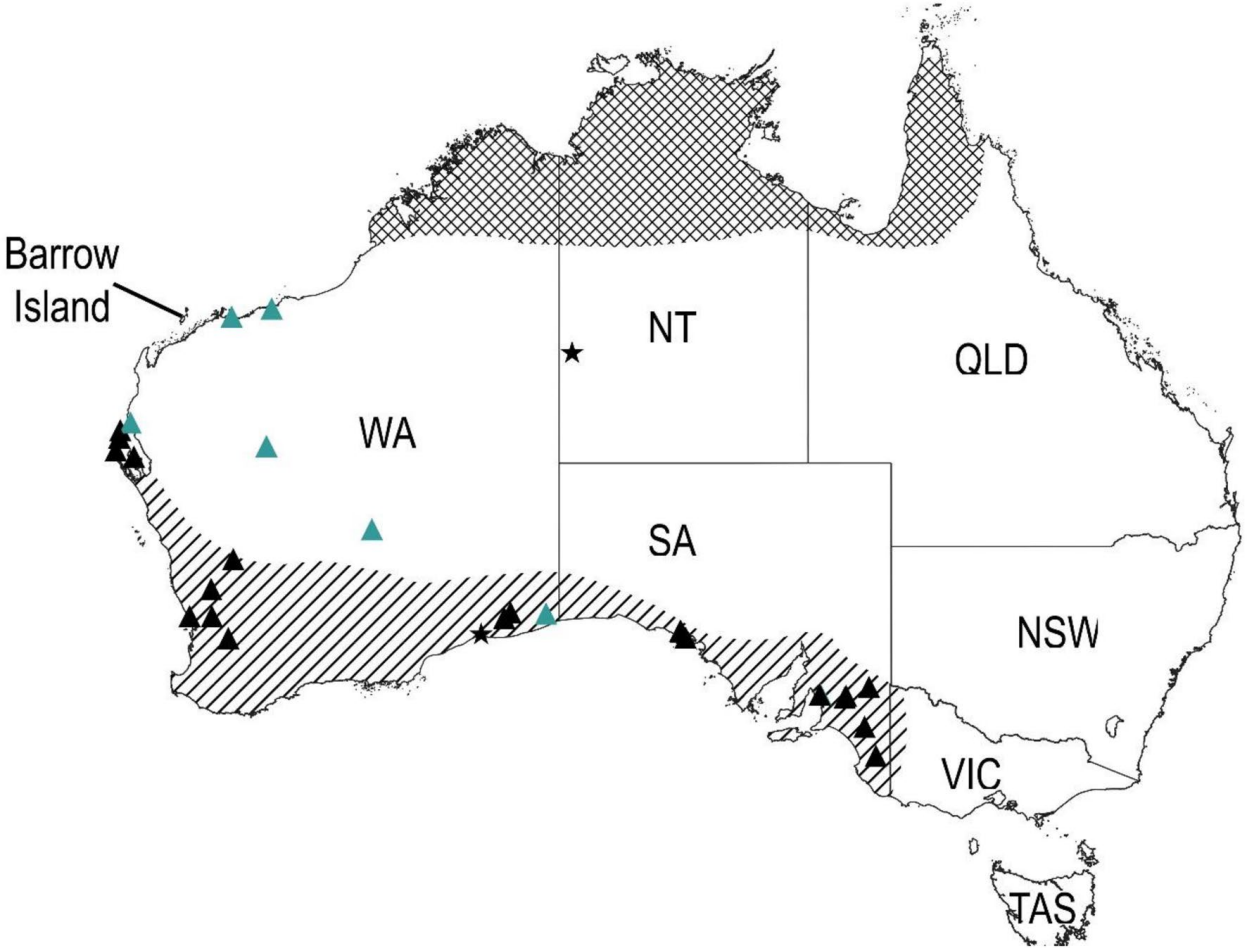
Current and historical distribution of banded hare-wallabies (*Lagostrophus fasciatus*), northern nail-tail wallabies (*Onychogalea unguifera*) and *Bettongia anhydra*. Black filled triangle: modern and subfossil occurrences of banded hare-wallabies; Green filled triangle: late nineteenth century sightings of banded hare-wallaby^38,66^; Filled star: modern and subfossil occurrences of *B. anhydra*^67^. Diagonal hatching shows the historically understood range of banded hare-wallabies and cross hatching shows the current range of northern nail-tail wallabies^68^. Figure generated with QGIS (v.3.22) (www.qgis.org).

Our study has identified the postcranial remains of at least two macropod species, banded hare-wallabies and northern nail-tail wallabies, which are no longer found in the Pilbara bioregion. Two astragali classified as banded hare-wallaby provide the first subfossil evidence for the distribution of this species north of the 24th parallel. These two specimens, one of which shows evidence of light localized burning, were identified from dense occupation deposits dated to the terminal Pleistocene (ca. 13–10 ka BP). Today the banded hare-wallaby is only found as relict populations on islands in Shark Bay, and its historical distribution has been thought to be restricted to dry temperate regions in southern Australia (Fig. 3). This species relies on thick scrub (commonly *Acacia*) for shelter during the day and emerges at night to browse on forbs and shrubs^70^. Shortridge reported historical sightings of the banded hare-wallaby from the late nineteenth century, as far north as Port Hedland and in the eastern Gascoyne and Murchison bioregions (Fig. 3)^38^. However, these reports have been disregarded in the literature due to the lack of specimens and potential for confusion with other hare-wallabies^66^. Our findings support the historical sightings reported in Shortridge and indicate that banded hare-wallabies likely inhabited the Pilbara from at least the terminal Pleistocene until recent times.

Two ankle bones belonging to the northern nail-tail wallaby were identified from deposits dated to the early Holocene (ca. 10 ka BP). Remains of this species have previously been reported from Holocene deposits in Boodie Cave and Noala and Hayne’s Caves on the neighbouring Montebello Islands^28,33^. Today, the northern nail-tail wallaby is found only in tropical woodland, north of the 500 mm isohyet (Fig. 3). This species also uses thick scrub to shelter during the day and emerges at night to feed on forbs, fruits and grasses^68^. To date, evidence for this species in the Pilbara has been restricted to Holocene deposits.

While banded hare-wallabies and northern nail-tail wallabies are today restricted far to the south and north of Boodie Cave, it is possible that these two species consistently occupied the arid subtropics and have broader ecological tolerances than are currently observed. Alternatively, their presence at Boodie Cave during the terminal Pleistocene and early Holocene could represent a pulse of increased macropod diversity in response to ameliorating environmental conditions after the Last Glacial Maximum (LGM). Regional paleoclimatic records indicate the Austral monsoon returned to the Pilbara region ca. 14,000 cal BP, bringing a period of warm, humid conditions following the intense aridity of the LGM^71^. Isotopic and micromorphological evidence from Boodie Cave support the regional pattern for wetter local conditions during the terminal Pleistocene and early Holocene than are seen today^72,73^. Anthracological examination of hearth fuels at Boodie Cave also indicates a localized increase in *Acacia*-dominated vegetation cover in association with these warm and humid conditions^33^. Paleontological research has demonstrated that, until recently the terrestrial fauna of northwest Australia was much more diverse^37,74,75^. However, no evidence for the banded-hare wallaby or northern nail-tail wallaby has yet been reported prior to the terminal Pleistocene. Increased humidity and vegetation cover at this time may have produced the required shelter and herbaceous feed for these two macropods that may not have been available during earlier periods of higher aridity. Additional paleozoological assemblages may provide further insight into the antiquity and longevity of these two species in the Pilbara. The co-occurrence of these two species on the Pilbara coast, which are now geographically distinct is indicative of the unique position of this bioregion at the junction of temperate and tropical climatic systems.

Four small and unusually shaped bettong ankle bones were identified from excavation units at Boodie Cave dated to immediately after the LGM, the terminal Pleistocene, and early and very late Holocene (Fig. 2a). Two distinct forms of bettong, a small and large form, have previously been reported from Holocene deposits at Noala and Haynes Cave^28^. At that time these morphotypes were interpreted as two co-existing subspecies of the burrowing bettong, however it was noted that further taxonomic resolution of this species was required. Subsequent research has identified *Bettongia anhydra*, as an additional arid zone bettong species previously misidentified as a subspecies of either the burrowing or brush-tailed bettong^67^. Closely related to the burrowing bettong, little is known about the former distribution of this recently extinct species (Fig. 3), although their desert-adapted cranial traits suggest this species may have once been widely distributed throughout the arid zone. As *B. anhydra* was smaller than the burrowing bettong, the four, small and unusually shaped bettong ankle bones in Boodie Cave potentially belong to this species.

## Conclusion

We have presented a quantitative method that can robustly classify marsupial postcranial bones, which will increase the suite of skeletal elements that can be used from archaeological faunal assemblages in Australasia. Understanding skeletal part representation plays an important role in interpreting past foraging behaviours. Therefore, enhancing our ability to identify postcrania to higher taxonomic levels is essential if we wish to move beyond the ‘laundry lists’ of species presence and absence and examine wider anthropological questions concerning diet breadth and human mobility^76^. The presence of banded hare-wallabies and northern nail-tail wallabies at Boodie Cave provides independent support for the regional patterns of paleoenvironmental and ecological change along the arid Pilbara coast during the terminal Pleistocene. Despite the small size of our archaeological sample, the distribution of macropod foot and ankle bones at Boodie Cave supports the wider regional patterns for increasing dietary diversity and occupation intensity and decreasing mobility of Indigenous communities along the Pilbara coastline^28,35,36^.

Our method has global implications for regions with extensive adaptive radiations. In Australasia, South America, Southeast Asia and the Neotropics, the ability to differentiate postcranial remains of closely related taxa would have a transformative effect on paleozoological research. The postcranial morphological similarities between closely related taxa means that examining taxonomic diversity in the absence of craniodental specimens is highly challenging. Morphometrics are increasingly being employed in Quaternary research to differentiate between postcranial specimens of morphologically similar wild animals such as rhea^18^, swiftlets^17^, fish^16,77^, and foxes^78^. However, the potential application of these methods to large and diverse faunal groups has yet to be fully realised.

Morphometric approaches towards the identification of paleozoological specimens are empirical and replicable between operators and can be applied across different faunal assemblages to answer both archaeological and paleontological research questions. These methods can be used to identify specimens, including potentially novel or extinct taxa, even in the absence of physical reference specimens. Open and collaborative access to osteometric data enhances the power of discriminant models for taxonomic classification. This is particularly important in regions with high extinction rates and where access to extensive reference collections may be limited or unequal between researchers.

## Supplementary Information

Supplementary Information.

## Data Availability

All data needed to evaluate the conclusions in the paper are present in the paper and/or the Supplementary Materials. Specimen catalogue details, raw measurement data and R code needed to reproduce the figures and tables are available at https://github.com/ErinMein/Morphometric_classification_kangaroo_bones.git.
