# Supplementary material for: Morphometric classification of kangaroo bones reveals paleoecological change in northwest Australia during the terminal Pleistocene
Source: Sci Rep. 2022 Oct 29;12:18245. doi: 10.1038/s41598-022-21021-w (PMC9617867; doi:10.1038/s41598-022-21021-w)
Supplement: Supplementary file 1 — Supplementary Information. [file 41598_2022_21021_MOESM1_ESM.pdf]

Morphometric classification of kangaroo bones reveals paleoecological change in northwest Australia during the terminal Pleistocene

Erin Mein[1*], **Tiina Manne**[1 2 3], **Peter Veth**[2 4], **Vera Weisbecker**[2 5]

[1] School of Social Science, University of Queensland, St Lucia. e.mein@uq.edu.au
[2] Australian Research Council Centre of Excellence for Australian Biodiversity and Heritage, University of Wollongong, Wollongong.
[3] Max Plank Institute for the Science of Human History, Jena, Germany.
[4] Archaeology, School of Social Sciences, University of Western Australia, Crawley.
[5] College of Science and Engineering, Flinders University, Bedford Park

Supplementary Note. Pes bone shape variation between macropod species

Large macropods

The main separation among astragali of large macropods is along Principal Component (PC) 2. This is driven by the breadth of the navicular facet, followed by differences in the proportions of the talocalcaneal facets on the plantar surface of the astragalus (Fig. S1). The astragali of agile wallaby, common wallaroo and the northern nail-tail wallaby exhibit a broad navicular facet, a shallow and narrow medial talocalcaneal facet and a short lateral talocalcaneal facet. In contrast red and grey kangaroo astragali have a deep and broad medial talocalcaneal facet and a longer lateral talocalcaneal facet.

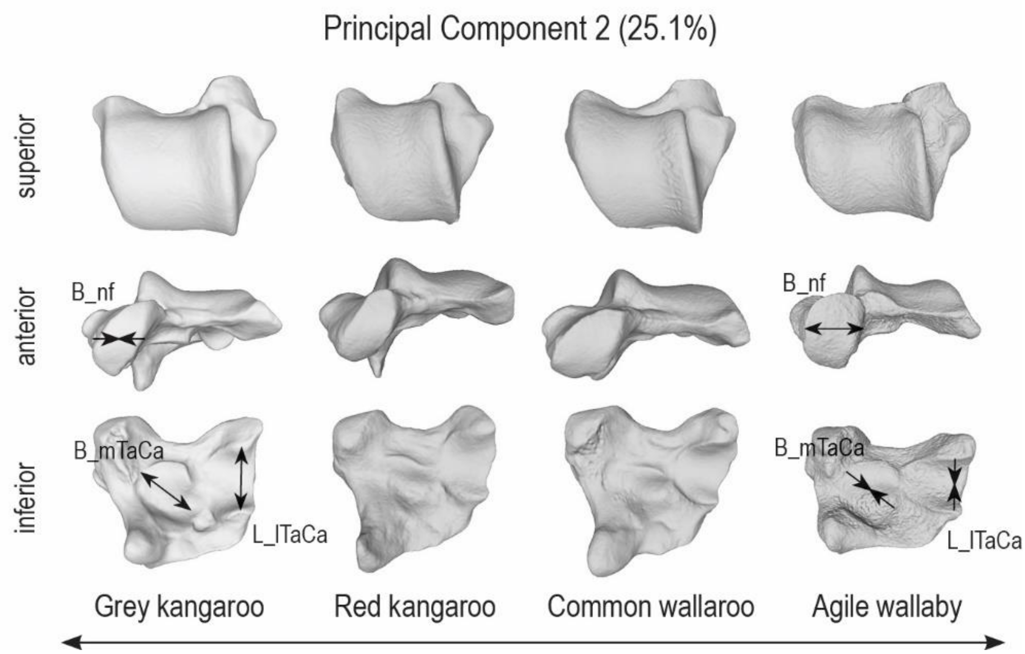

Figure S1. Proportional shape change between astragali of large macropods. Arrows indicate the direction of measurement change between taxa.

Calcanea of agile wallaby and northern nail-tail wallaby are separated from the other large macropods along PC1 by a more deeply stepped calcaneocuboid articulation and a broader sustentaculum tali for passage of the flexor digitorum longus (Fig. S2). Grey and red kangaroos and the wallaroos have a broader tuber calcanei and a longer lateral calcaneotalar facet. Red and grey kangaroos are separated from the wallaroos along PC2 by a more deeply stepped calcaneocuboid articulation and more slender tuber calcanei.

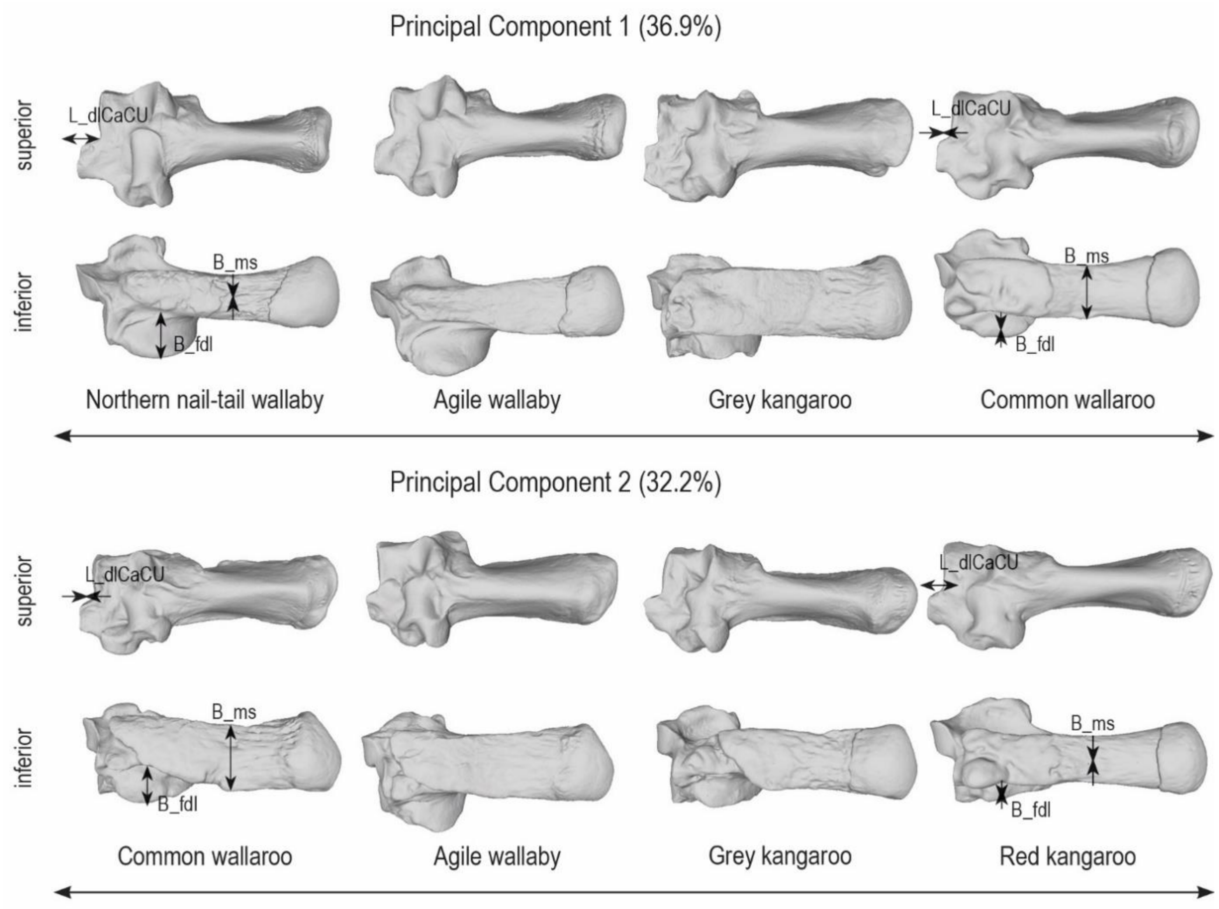

Supplementary Figure S2. Shape variation between calcanea of large macropods.

Fourth metatarsal shape of large macropods overlaps substantially, although some difference can be observed along PC1 associated with metatarsal length and robusticity of the metatarsal shaft. Red and grey kangaroos have longer, more mediolaterally slender metatarsals, while wallaroos, agile wallabies and northern nail-tail wallabies have shorter, more robust metatarsals (Fig. S3).

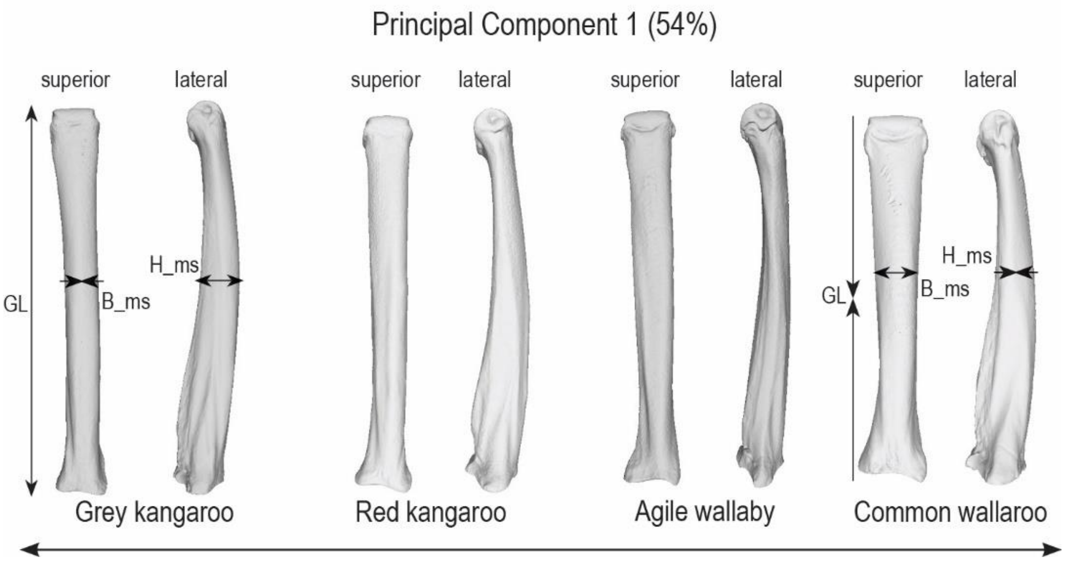

Supplementary Figure S3. Shape variation between fourth metatarsals of large macropods.

Medium macropods

The medium sized rock-wallaby astragali are separated from spectacled hare-wallabies and the nail-tail wallabies along PC1 by a longer lateral talocalcaneal facet and a broad navicular facet (Fig. S4). The astragali of spectacled hare-wallabies and nail-tail wallabies have a deeper medial talocalcaneal facet and broader trochlear crests than rock-wallabies. While astragali of both species of nail-tail wallaby are similar in shape to spectacled hare-wallabies the former are substantially larger in size.

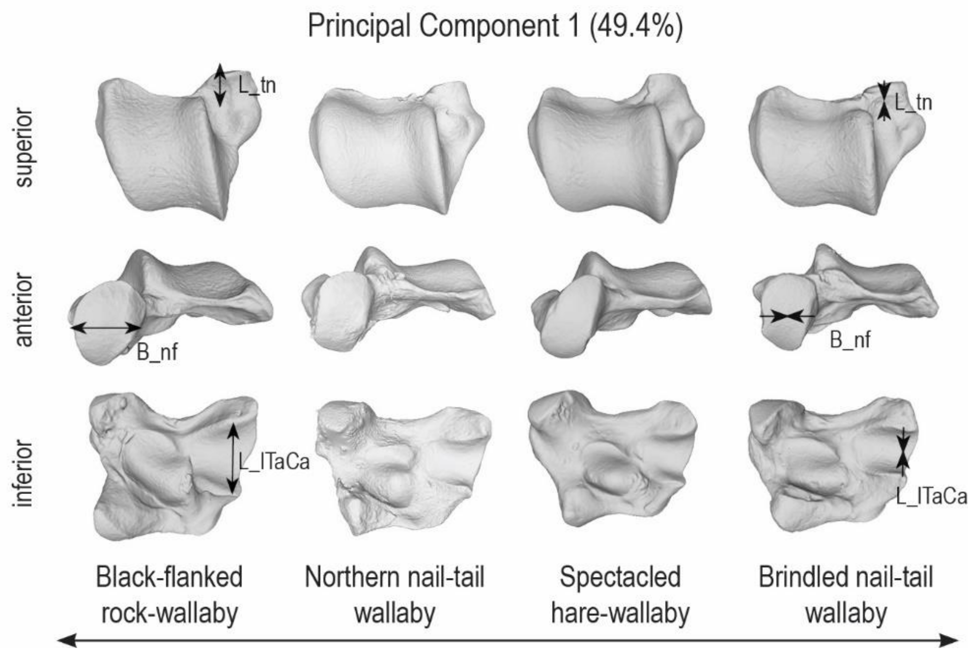

Supplementary Figure S4. Shape differences between astragali of medium macropods. Arrows indicate direction of measurement change between genera.

Medium sized rock-wallaby calcanea are separated from spectacled hare-wallabies and nail-tail wallabies along PC1 by a longer lateral calcaneotalar facet and a broader tuber calcanei (Fig. S5). In contrast, spectacled hare-wallabies and nail-tail wallabies have more deeply stepped calcaneocuboid articulation and a deeper medial sustentaculum tali.

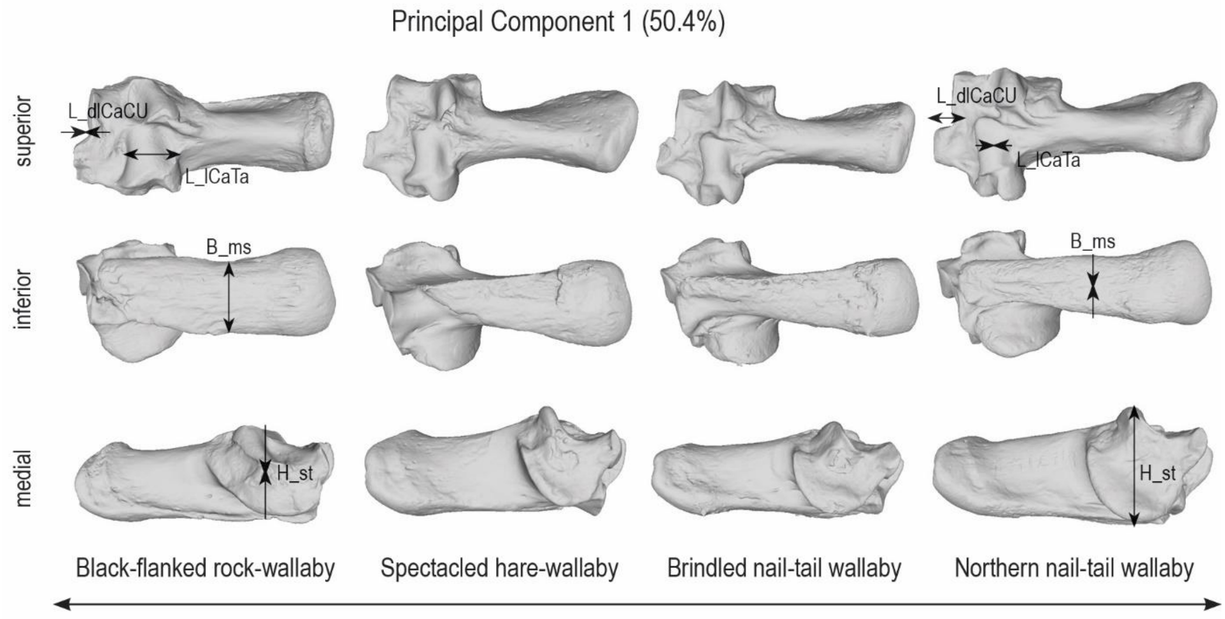

Supplementary Figure S5. Shape variation between calcanea of medium macropods. Arrows indicate direction of measurement change between genera.

Rock-wallabies are differentiated from spectacled hare-wallabies along PC1 by comparatively short and mediolaterally broad fourth metatarsals (Fig. S6). Metatarsal shape of the two species nail-tail wallabies does not overlap in the main variation of the PCA. Northern nail-tail wallabies exhibit more slender metatarsals than bridled nail-tail wallabies which are very similar in shape to those of rock-wallabies but have a dorsoventrally taller shaft.

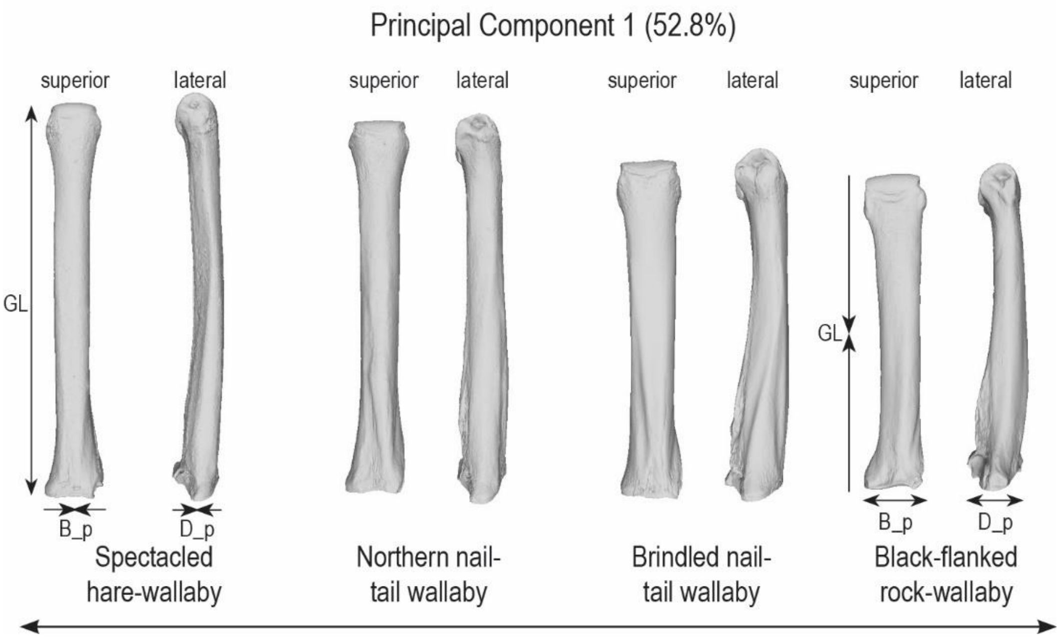

Supplementary Figure S6. Shape variation between fourth metatarsals of medium macropods. Arrows indicate direction of measurement change between genera.

Small macropods

The astragali of bettongs have a narrower lateral talocalcaneal facet, broader medial talocalcaneal facet and broader navicular facet than rufous and spectacled hare-wallabies (Fig. S7). Banded hare-wallaby astragali are intermediate in shape between the bettongs and other hare-wallabies in the main variation of the PCA and share similar features with both. Astragali of juvenile small macropods tend to score negatively along PC2 which is strongly associated with a longer talar neck.

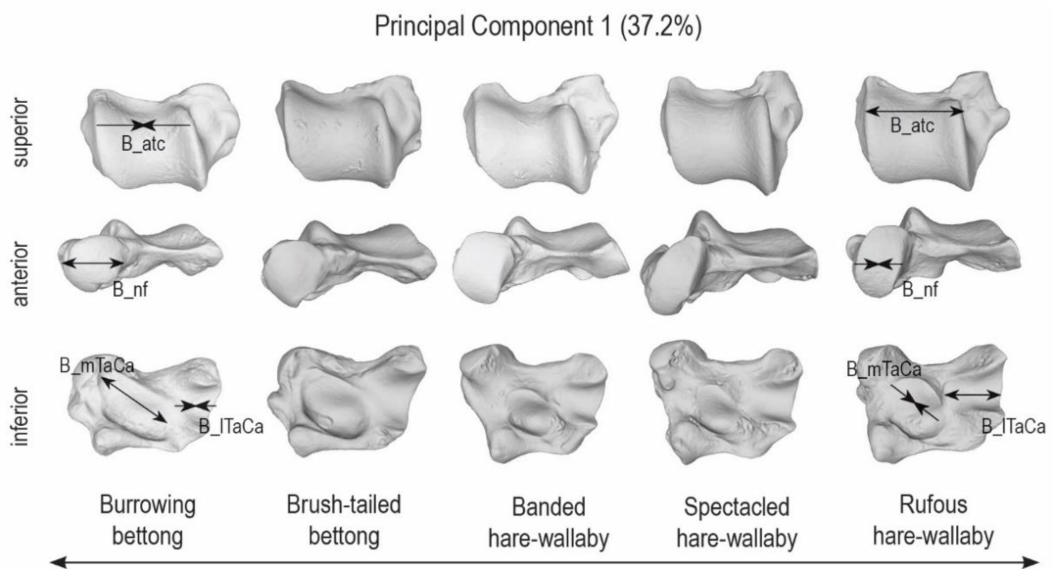

Supplementary Figure S7. Shape variation between astragali of small macropods. Arrows indicate direction of measurement change between genera.

Bettong calcanea are separated from the other small macropods along PC1 by a narrow tuber calcanei, and broad dorsolateral calcaneocuboid articulation and broad medial calcaneotalar facet (Fig. S8). Conversely hare-wallabies and the Nabarlek have a broad tuber calcanei, a narrow medial calcaneotalar facet and long medial process on the sustentaculum tali. The shape of hare-wallaby calcanea vary from the Nabarlek along PC2 with the latter exhibiting a longer lateral calcaneotalar facet and shorter calcaneocuboid step.

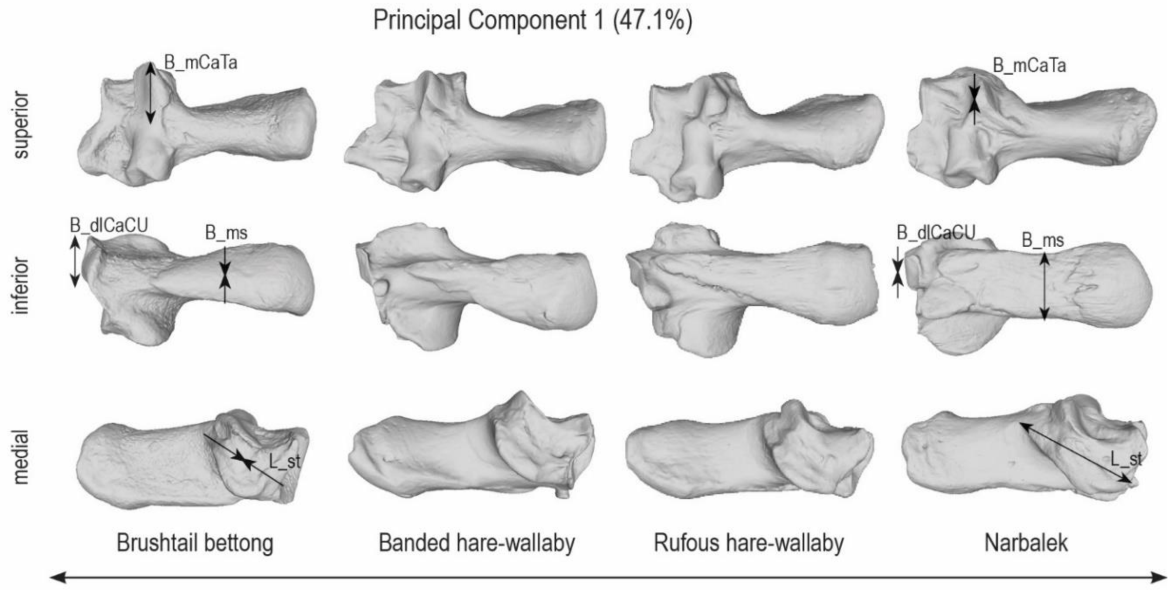

Supplementary Figure S8. Shape variation between calcanea of small macropods. Arrows indicate direction of measurement change between genera.

Bettong and banded-hare wallaby fourth metatarsal shape overlaps entirely in the main variation of the PCA and are visually very similar in shape (Fig. S9). Rufous hare-wallaby metatarsals can be differentiated from other small macropods by their longer and more slender shape. Narbalek metatarsals are intermediate in shape to the rufous hare-wallaby and bettongs. Although proportionally similar, the proximal end of bettong fourth metatarsals is distinctive, exhibiting a ‘L’ shaped articulation while other small macropods have a more triangular proximal articulation (Fig. S9).

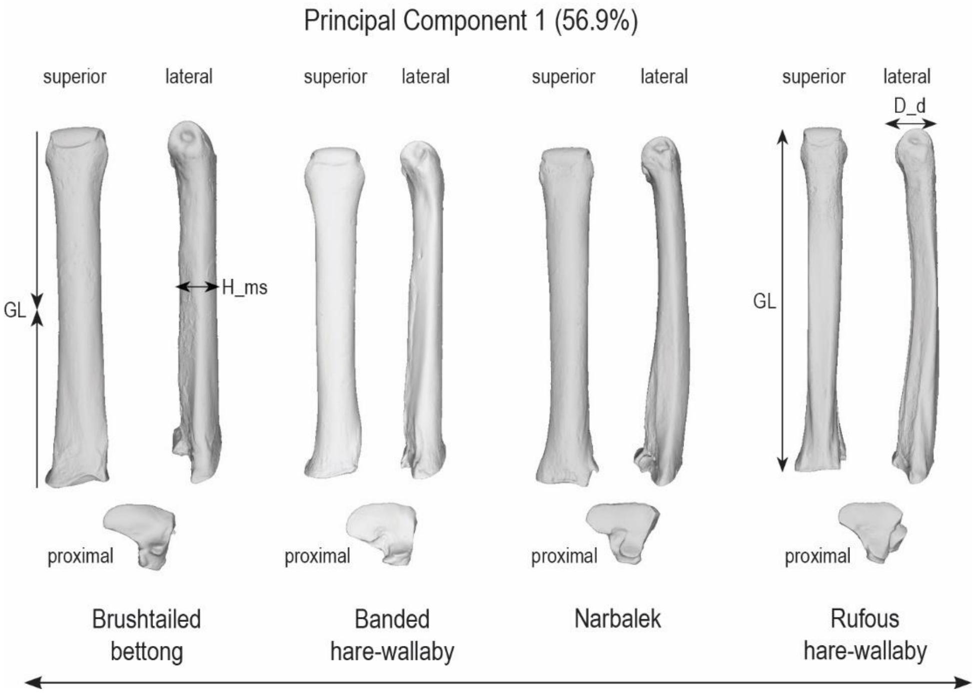

Supplementary Figure S9 Shape variation between fourth metatarsals of small macropods. Arrows indicate direction of measurement change between genera.

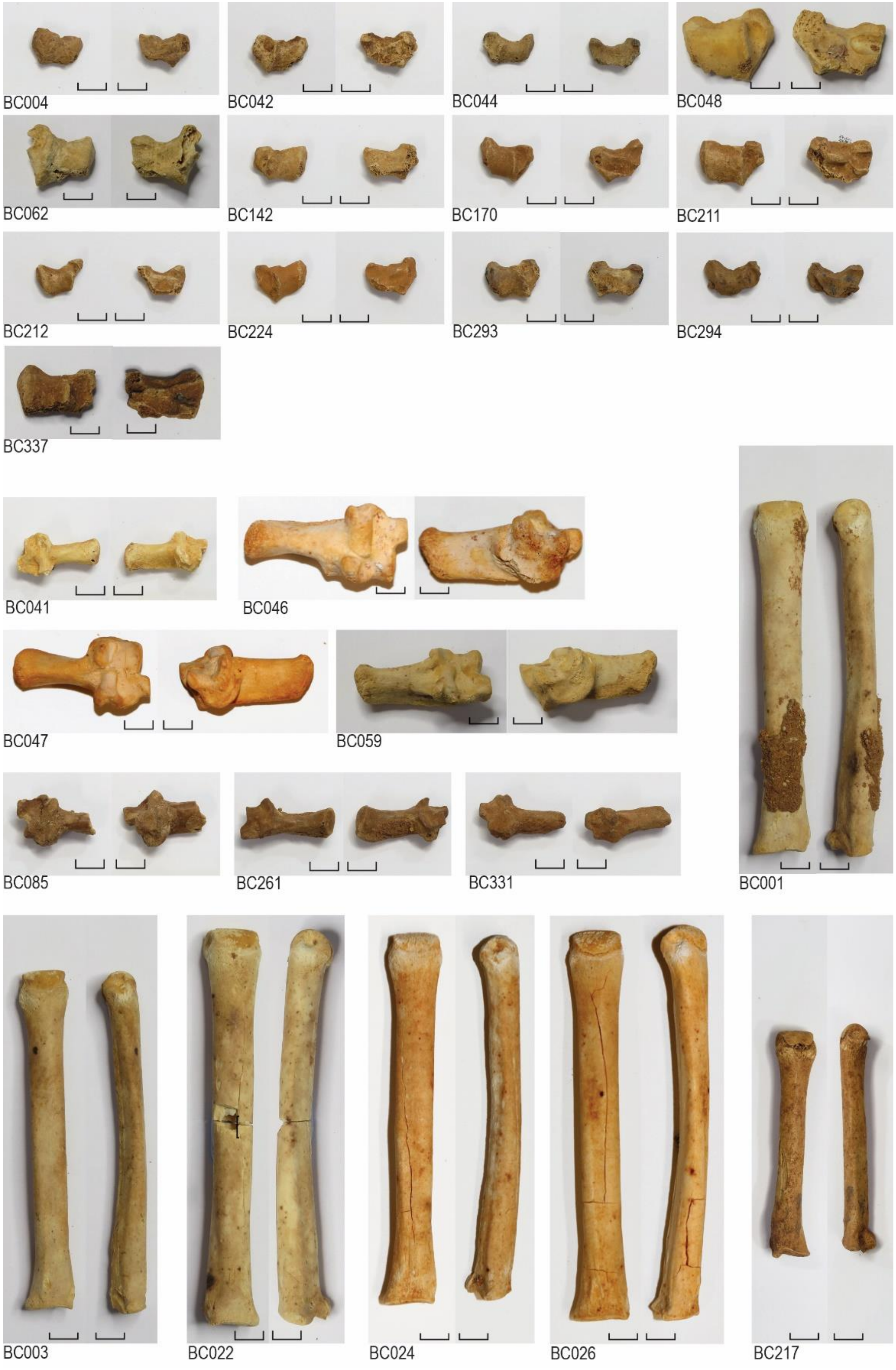

Supplementary Figure S10. Archaeological specimens from Boodie Cave examined in this study. (Top) astragali superior (L) and inferior views (R); (Middle) calcanea superior (L) and medial (R) views; (Bottom) fourth metatarsals superior (L) and lateral (R) views.

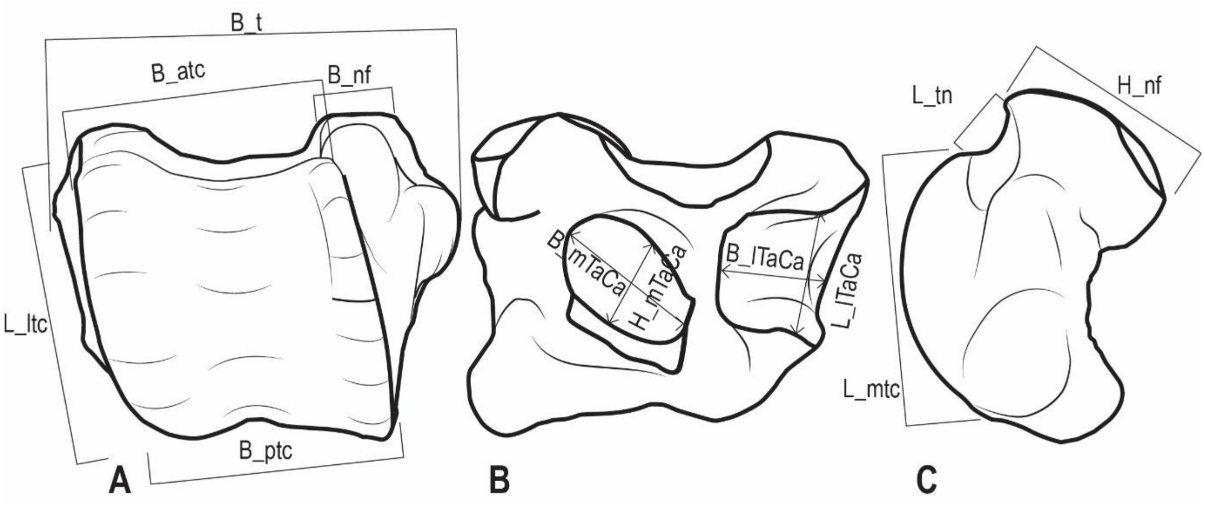

Supplementary Figure S11. Left macropod astragalus showing measurements used in this study. (A) superior view, **(B)** inferior view, **(C)** medial view.

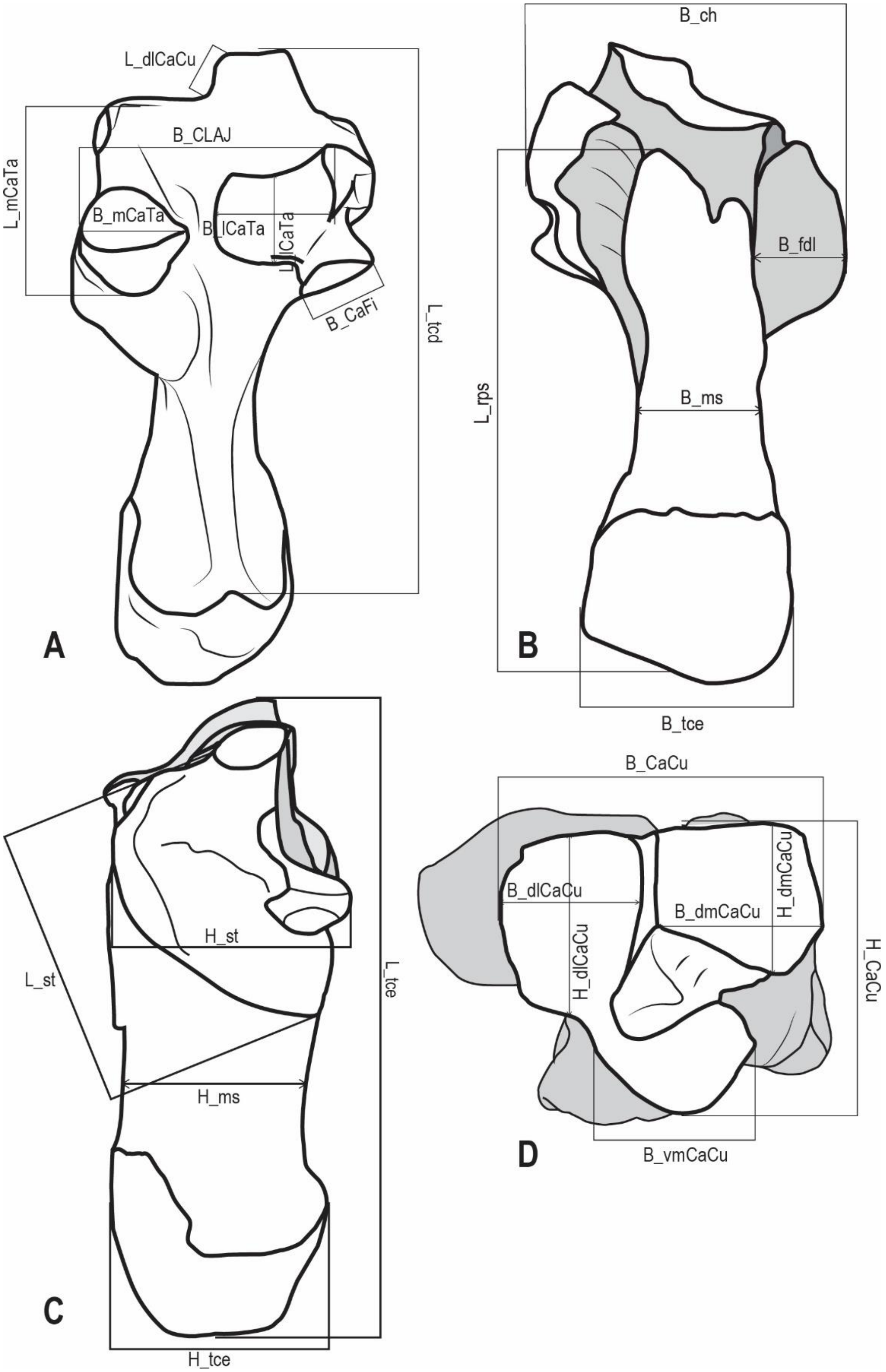

Supplementary Figure S12. Right macropod calcaneus, showing measurements used in this study. (A) superior view, **(B)** inferior view, **(C)** medial view, **(D)** anterior view.

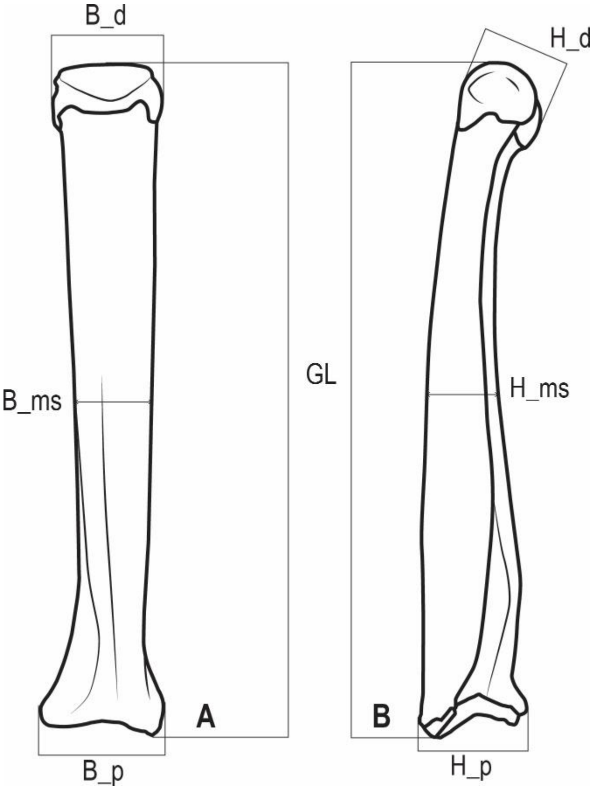

Supplementary Figure S13. Left macropod fourth metatarsal showing measurements used in this study. (A) superior view, **(B)** medial view.

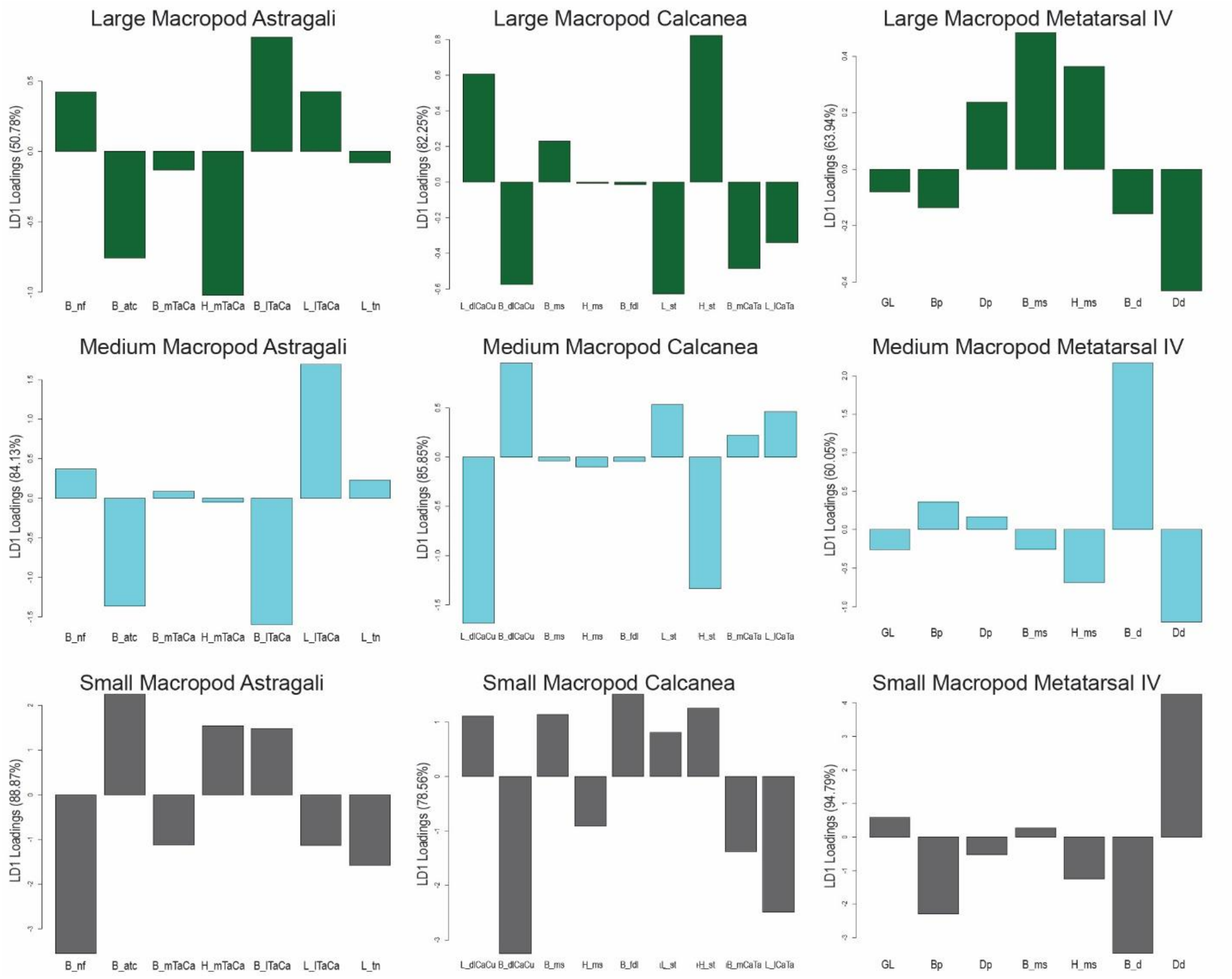

Supplementary Figure S14. Loadings for linear discriminant 1 on three macropod pes bones. top) large macropod astragali (left), calcanea (middle) & fourth metatarsals (right); **middle)** medium macropod astragali (left), calcanea (middle) & fourth metatarsals (right); **bottom)** small macropod astragali (left), calcanea (middle) & fourth metatarsals (right)

Supplementary Table S1. Quantity of skeletal elements by species used as the training dataset.

Taxa	Astragali	Calcanea	Metatarsal IV
Agile wallaby *(Notamacropus agilis)*	13	13	9
Antilopine wallaroo *(Osphranter antilopinus)*	6	5	5
Banded hare-wallaby *(Lagostrophus fasciatus*	12	10	6
Black-flanked rock-wallaby *(Petrogale lateralis)*	17	16	14
Black wallaroo *(Osphranter bernardus)*	5	5	4
Bridled nail-tail wallaby *(Onychogalea fraenata)*	12	13	7
Brushtail bettong (*Bettongia penicillata)*	14	14	10
Burrowing bettong *(Bettongia lesueur)*	10	8	5
Common Wallaroo/Euro *(Osphranter robustus)*	19	19	17
Nabarlek *(Petrogale concinna)*	3	3	3
Northern nail-tail wallaby *(Onychogalea unguifera)*	10	11	7
Red kangaroo *(Osphranter rufus)*	16	14	14
Rothschild's rock-wallaby *(Petrogale rothschildi)*	5	5	2
Rufous hare-wallaby *(Lagorchestes hirsutus)*	18	17	14
Short-eared rock-wallaby *(Petrogale brachyotis)*	3	3	3
Spectacled hare-wallaby *(Lagorchestes conspicillatus)*	11	13	10
Western grey kangaroo *(Macropus fuliginosus)*	10	11	12
**Total**	**184**	**180**	**142**

Supplementary Table S2. Archaeological specimens from Boodie Cave examined in this study. (SQ) Excavation square, **(XU)** Excavation Unit, **(SU)** Stratigraphic Unit.

Specimen ID	SQ	XU	SU	Skeletal element	Body Side
BC041	F101	400	1	calcaneus	L
BC003	G101	533	2	fourth metatarsal	L
BC026	G101	534	3	fourth metatarsal	L
BC001	G101	535	3	fourth metatarsal	L
BC024	G101	535	3	fourth metatarsal	L
BC022	G101	536	3	fourth metatarsal	R
BC217	B111	306	3	fourth metatarsal	R
BC337	A106	29	3	astragalus	L
BC261	A107	13	3	calcaneus	L
BC331	A106	33	3	calcaneus	R
BC062	E101	606	4	astragalus	R
BC059	E101	606	4	calcaneus	R
BC142	G100	509	4/5	astragalus	R
BC047	F101	408	5	calcaneus	R
BC048	F101	408	5	astragalus	L
BC046	F101	408	5	calcaneus	L
BC004	G101	538	5	astragalus	R
BC211	A103	249	5	astragalus	L
BC212	A103	249	5	astragalus	L
BC044	F101	409	5	astragalus	R
BC085	E101	610	5	calcaneus	R
BC170	A103	255	5	astragalus	L
BC042	F101	415	6	astragalus	R
BC224	A102	227	6	astragalus	R
BC293	A102	264	7	astragalus	L
BC294	A102	264	7	astragalus	L

Supplementary Table S3. Description of measurements taken on macropod astragali.
(*) Denotes measurements included after redundancy analysis.

Measurement	Description
B_nf *	Mediolateral breadth of navicular facet
H_nf	Dorsoventral height of navicular facet
L_tn *	Anteroposterior length of talar neck
L_mtc	Anteroposterior length medial trochlear crest
L_ltc	Anteroposterior length of lateral trochlear crest
B_atc *	Mediolateral breadth of trochlear crests (anteriorly)
B_ptc	Mediolateral breadth of trochlear crests (posteriorly)
B_t	Greatest mediolateral breadth of the astragalus
B_mTaCa *	Greatest mediolateral breadth of medial talocalcaneal facet, measured perpendicular to direction of joint movement.
H_mTaCa *	Greatest dorsoventral height of anterior surface of the medial talocalcaneal facet, measured parallel to direction of joint movement.
B_lTaCa *	Greatest mediolateral breadth of lateral talocalcaneal facet, measured at the maximum curvature of the facet.
L_lTaCa *	Anteroposterior length of the lateral talocalcaneal facet, measured at the point of maximum curvature of the anterior and posterior margins of the facet.

Supplementary Table S4. Description of measurements taken on macropod calcanea.
(*****) Denotes measurements included after redundancy analysis.

Measurement	Description
L_dlCaCu *	Anteroposterior length of the dorsolateral calcaneocuboid facet.
B_dlCaCu *	Greatest mediolateral breadth of the dorsolateral calcaneocuboid facet.
H_dlCaCu	Greatest dorsoventral height of the dorsolateral calcaneocuboid facet.
B_dmCaCu	Greatest mediolateral breadth of the dorsomedial calcaneocuboid facet.
H_dmCaCu	Greatest dorsoventral height of the dorsomedial calcaneocuboid facet.
B_CaCu	Breadth of calcaneocuboid articulation
H_CaCu	Height of calcaneocuboid articulation
L_tcd	Length of calcaneus from dorsolateral calcaneocuboid facet to epiphysis suture
H_tce	Height of epiphysis
B_tce	Breadth of epiphysis
L_rps	Anteroposterior length of the rugrose plantar surface, measured on the lateral side, parallel to tuber calcanei.
B_ms *	Mediolateral breadth of the tuber calcanei, measured at the midpoint of the rugrose plantar surface.
H_ms *	Dorsoventral height of the tuber calcanei, measured at the same point as the B_ms.
B_ch	Greatest mediolateral breadth of the sustentaculum tali
B_fdl *	Greatest breadth of the medial projection of the sustentaculum tali beyond the wall of the tuber calcanei, forming a sulcus for the passage of the flexor digitorum longus.
L_st *	Greatest anteroposterior length of the semi-circular crest of the medial sustentaculum tali.
H_st *	Greatest dorsoventral height of the medial sustentaculum tali, measured from the top of the medial calcaneotalar facet to the underside of the semi-circular crest of the medial sustentaculum tali.
L_mCaTa	Anteroposterior length of medial calcaneotalar facet, including the site of attachment for the ligamentum cervicis tali.
B_mCaTa *	Mediolateral breadth of the medial calcaneotalar facet.
L_lCaTa *	Anteroposterior length of the lateral calcaneotalar facet.
B_lCaTa	Mediolateral breadth of the lateral calcaneotalar facet.
B_CLAJ	Breadth of the continuous lower ankle joint
B_CaFi	Breadth of calcaneofibular articulation
H_CaFi	Height of calcaneofibular articulation, measured perpendicular to B_CaFi

Supplementary Table S5. Description of measurements taken on macropod fourth metatarsals.

(*****) Denotes measurements included after redundancy analysis.

**Measurement**	**Description**
GL *	Greatest anteroposterior length of metatarsal.
B_p *	Greatest mediolateral breadth of proximal end of the metatarsal.
H_p *	Greatest dorsoventral height of proximal end of the metatarsal.
B_ms *	Mediolateral breadth of the shaft at half the length of the metatarsal.
H_ms *	Dorsoventral height of the shaft at the same point as B_ms.
B_d *	Greatest mediolateral breadth of the distal epiphysis of the metatarsal.
H_d *	Greatest dorsoventral height of the distal epiphysis of the metatarsal.

Supplementary Table S6. Mean standard deviation of repeat measurements proportional to repeat measurement mean (s / $\bar{x}$) and intraclass correlation coefficients (ICC3) for each measurement (r). All intraclass correlations are significant at <0.001.

Variable	Large Macropods		Medium Macropods		Small Macropods	
	**s / $\bar{x}$**	**r**	**s / $\bar{x}$**	**r**	**s / $\bar{x}$**	**r**
Astragali						
B_nf	0.017	0.991	0.022	0.975	0.023	0.960
H_nf	0.007	0.998	0.007	0.996	0.012	0.967
L_tn	0.028	0.974	0.042	0.929	0.045	0.895
L_mtc	0.005	0.999	0.01	0.987	0.011	0.986
L_ltc	0.011	0.994	0.014	0.986	0.018	0.963
B_atc	0.011	0.996	0.016	0.983	0.017	0.946
B_ptc	0.012	0.992	0.02	0.965	0.018	0.947
B_t	0.005	0.999	0.005	0.997	0.005	0.992
B_mTaCa	0.015	0.989	0.023	0.956	0.026	0.950
H_mTaCa	0.021	0.971	0.031	0.948	0.027	0.915
B_lTaCa	0.015	0.990	0.026	0.965	0.03	0.936
L_lTaCa	0.014	0.993	0.024	0.976	0.022	0.958
Calcanea						
L_dlCaCu	0.032	0.970	0.041	0.960	0.034	0.893
B_dlCaCu	0.016	0.989	0.019	0.969	0.018	0.962
H_dlCaCu	0.016	0.993	0.024	0.975	0.024	0.980
B_dmCaCu	0.009	0.996	0.019	0.981	0.020	0.961
H_dmCaCu	0.013	0.996	0.021	0.984	0.029	0.891
B_CaCu	0.006	0.998	0.009	0.995	0.006	0.984
H_CaCu	0.008	0.998	0.011	0.992	0.010	0.962
L_tcd	0.004	0.999	0.007	0.996	0.007	0.994
H_tce	0.007	0.998	0.015	0.982	0.013	0.978
B_tce	0.005	0.999	0.008	0.992	0.010	0.988
L_rps	0.004	0.999	0.006	0.997	0.009	0.996
B_ms	0.009	0.998	0.017	0.986	0.032	0.953
H_ms	0.007	0.999	0.012	0.993	0.012	0.970
B_ch	0.007	0.996	0.007	0.995	0.010	0.982
B_fdl	0.016	0.989	0.023	0.982	0.024	0.959
L_st	0.009	0.997	0.015	0.986	0.021	0.969
H_st	0.011	0.994	0.015	0.994	0.015	0.972
L_mCaTa	0.012	0.993	0.015	0.984	0.016	0.959
B_mCaTa	0.016	0.989	0.022	0.962	0.028	0.910
L_lCaTa	0.016	0.994	0.028	0.969	0.028	0.891
B_lCaTa	0.017	0.991	0.022	0.975	0.034	0.873
B_CLAJ	0.007	0.997	0.010	0.992	0.012	0.945
H_CaFi	0.020	0.986	0.028	0.960	0.039	0.857
B_CaFi	0.018	0.992	0.034	0.960	0.029	0.903
Fourth Metatarsal						
GL	0.001	1.000	0.001	1.000	0.001	1.000
Bp	0.005	0.999	0.007	0.996	0.009	0.970
Dp	0.006	0.998	0.008	0.996	0.008	0.973
B_ms	0.009	0.995	0.007	0.997	0.009	0.983
H_ms	0.015	0.990	0.012	0.994	0.012	0.965
Bd	0.005	0.999	0.004	0.999	0.002	0.998
Dd	0.005	0.999	0.009	0.995	0.009	0.979

Supplementary Table S7. Multivariate analysis of variance on large macropod pes bone shape.

Principal component scores comprising 95% of total shape variation were used as a proxy for bone shape. Groups with < n-2 observations (where n is the number of principal components) were not tested. Bold indicates a significant p-value (<0.01).

Taxa	MANOVA	df1	df2	F	p
	Astragali				
**All large macropods**	**Shape ~ Age**	**5**	**62**	**9.376**	**<0.001**
Agile wallaby	Shape ~ Size	5	7	0.965	0.498
	Shape ~ Age	5	7	5.994	0.018
	Shape ~ Sex	5	5	0.570	0.724
***Osphranter* species**	**Shape ~ Age**	**5**	**32**	**4.154**	**0.005**
Common wallaroo	Shape ~ Island vs Mainland Subspecies	5	13	1.992	0.147
	Shape ~ Size	5	13	2.573	0.079
	Shape ~ Age	5	9	2.178	0.146
Red kangaroo	Shape ~ Size	5	10	2.482	0.104
	Shape ~ Age	5	7	0.549	0.736
	Shape ~ Sex	5	5	0.662	0.669
Western grey kangaroo	Shape ~ Size	5	4	3.242	0.139
	Shape ~ Age	5	4	3.885	0.106
	Calcanea				
**All large macropods**	**Shape ~ Age**	**6**	**61**	**9.459**	**<0.001**
Agile wallaby	Shape ~ Size	6	6	2.327	0.164
	Shape ~ Age	6	6	7.002	0.016
	Shape ~ Sex	6	4	1.500	0.362
***Osphranter* species**	**Shape ~ Age**	**6**	**29**	**6.235**	**<0.001**
Common wallaroo	Shape ~ Subspecies (Island vs Mainland)	6	12	3.468	0.032
	Shape ~ Size	6	12	3.988	0.02
	Shape ~ Age	6	9	1.015	0.472
Red kangaroo	Shape ~ Size	6	7	2.793	0.103
	Shape ~ Age	6	3	1.571	0.382
	Shape ~ Sex	6	3	0.467	0.804
Western grey kangaroo	Shape ~ Size	6	4	6.144	0.05
	Shape ~ Age	6	3	2.652	0.227
	Fourth metatarsals				
**All large macropods**	**Shape ~ Age**	**5**	**52**	**7.463**	**<0.001**
Agile wallaby	Shape ~ Size	5	3	7.707	0.062
	Shape ~ Age	5	3	0.576	0.725
	Shape ~ Sex	5	3	2.348	0.257
***Osphranter* species**	**Shape ~ Age**	**5**	**26**	**5.388**	**0.002**
**Common wallaroo**	**Shape ~ Subspecies (Island vs Mainland)**	**5**	**11**	**7.255**	**0.003**
	**Shape ~ Size**	**5**	**11**	**7**	**0.004**
	Shape ~ Age	5	9	0.375	0.854
Red kangaroo	Shape ~ Size	5	8	1.86	0.207
	Shape ~ Age	5	2	40.804	0.024
	Shape ~ Sex	5	4	5.665	0.059
Western grey kangaroo	Shape ~ Size	5	6	0.992	0.493
	Shape ~ Age	5	5	1.747	0.278
	Shape ~ Sex	5	1	6.074	0.298

Supplementary Table S8. Multivariate analysis of variance on medium macropod pes bone shape.

Principal component scores comprising 95% of total shape variation were used as a proxy for bone shape. Groups with < n-2 observations (where n is the number of principal components) were not tested. Bold indicates significant p-value (<0.01).

Taxa	MANOVA	df1	df2	F	p
	Astragali				
All medium macropods	Shape ~ Age	5	34	2.155	0.082
Onychogalea species	Shape ~ Age	5	7	1.641	0.266
Bridled nail-tail wallaby	Shape ~ Size	5	6	1.076	0.457
Northern nail-tail wallaby	Shape ~ Size	5	4	1.866	0.283
	Shape ~ Age	5	1	2.308	0.461
	Shape ~ Sex	5	1	1.407	0.562
Petrogale species	Shape ~ Age	5	14	1.122	0.393
Black-flanked rock-wallaby	Shape ~ Size	5	11	3.553	0.037
	Shape ~ Age	5	7	0.645	0.675
Spectacled hare-wallaby	Shape ~ Size	5	5	4.81	0.055
	Shape ~ Age	5	1	2.607	0.437
	Calcanea				
**All medium macropods**	**Shape ~ Age**	**6**	**37**	**3.609**	**0.006**
Onychogalea species	Shape ~ Age	6	8	2.952	0.08
Bridled nail-tail wallaby	Shape ~ Size	6	6	1.489	0.32
Northern nail-tail wallaby	Shape ~ Size	6	4	1.617	0.334
	Shape ~ Age	6	2	2.517	0.311
	Shape ~ Sex	6	1	9.898	0.239
Petrogale species	Shape ~ Age	6	13	0.975	0.479
Black-flanked rock-wallaby	Shape ~ Size	6	9	0.394	0.866
	Shape ~ Age	6	6	2.538	0.141
**Spectacled hare-wallaby**	**Shape ~ Size**	**6**	**6**	**8.321**	**0.01**
	Shape ~ Age	6	2	0.135	0.976
	Fourth metatarsals				
All medium macropods	Shape ~ Age	5	26	1.246	0.317
Onychogalea species	Shape ~ Age	5	2	1.974	0.37
Bridled nail-tail wallaby	Shape ~ Size	5	1	1284.631	0.021
Northern nail-tail wallaby	Shape ~ Size	5	1	1351.283	0.021
Petrogale species	Shape ~ Age	5	12	2.19	0.123
Black-flanked rock-wallaby	Shape ~ Size	5	8	2.178	0.157
	Shape ~ Age	5	7	2.783	0.107
Spectacled hare-wallaby	Shape ~ Size	5	4	0.635	0.688

Supplementary Table S9. Multivariate analysis of variance on small macropod pes bone shape.

Principal component scores comprising 95% of total shape variation were used as a proxy for bone shape. Groups with < n-2 observations (where n is the number of principal components) were not tested. Bold indicates significant p-value (<0.01).

Taxa	MANOVA	df1	df2	F	p
	Astragali				
All small macropods	Shape ~ Age	**6**	56	2.188	0.058
Banded hare-wallaby	Shape ~ Size	**6**	5	0.764	0.628
	Shape ~ Age	**6**	5	0.527	0.771
Bettongia species	Shape ~ Age	6	16	0.691	0.66
Brush-tailed bettong	Shape ~ Size	6	7	3.216	0.076
	Shape ~ Age	6	7	0.702	0.659
	Shape ~ Sex	6	4	0.94	0.55
Burrowing bettong	Shape ~ Size	6	3	5.493	0.095
	Shape ~ Age	6	2	0.251	0.921
Lagorchestes species	Shape ~ Age	6	18	1.37	0.279
**Rufous hare-wallaby**	**Shape ~ Island vs Mainland Subspecies**	**6**	**11**	**21.982**	**<0.001**
	Shape ~ Size	6	11	1.989	0.153
	Shape ~ Age	6	11	1.437	0.285
	Shape ~ Sex	6	5	1.136	0.454
	Calcanea				
All small macropods	Shape ~ Age	6	44	1.638	0.159
Banded hare-wallaby	Shape ~ Size	6	3	6.918	0.071
	Shape ~ Age	6	3	0.864	0.60
Bettongia species	Shape ~ Age	6	14	0.815	0.576
Brush-tailed bettong	Shape ~ Size	6	7	0.455	0.822
	Shape ~ Age	6	7	0.601	0.724
	Shape ~ Sex	6	4	1.066	0.498
Burrowing bettong	Shape ~ Size	6	1	15.644	0.191
**Rufous hare-wallaby**	**Shape ~ Subspecies (Island vs Mainland)**	**6**	**10**	**5.580**	**0.009**
	Shape ~ Size	6	10	1.035	0.457
	Shape ~ Age	6	10	1.999	0.159
	Shape ~ Sex	6	5	1.899	0.249
	Fourth metatarsals				
**All small macropods**	**Shape ~ Age**	**5**	**32**	**3.896**	**0.007**
Brush-tailed bettong	Shape ~ Size	5	4	0.293	0.894
	Shape ~ Sex	5	2	1.556	0.435
Rufous hare-wallaby	Shape ~ Sub-Species (Island vs Mainland)	5	8	2.852	0.091
	Shape ~ Size	5	8	1.573	0.271
	Shape ~ Age	5	8	0.663	0.662
	Shape ~ Sex	5	5	2.212	0.202
